# Myeloid cells promote interferon signaling-associated deterioration of the hematopoietic system

**DOI:** 10.1038/s41467-022-35318-x

**Published:** 2022-12-10

**Authors:** Jacqueline Feyen, Zhen Ping, Lanpeng Chen, Claire van Dijk, Tim V. D. van Tienhoven, Paulina M. H. van Strien, Remco M. Hoogenboezem, Michiel J. W. Wevers, Mathijs A. Sanders, Ivo P. Touw, Marc H. G. P. Raaijmakers

**Affiliations:** Department of Hematology, Erasmus MC Cancer Institute, 3015CN Rotterdam, the Netherlands

**Keywords:** Myelopoiesis, Haematopoietic stem cells, Neutrophils, Interferons

## Abstract

Innate and adaptive immune cells participate in the homeostatic regulation of hematopoietic stem cells (HSCs). Here, we interrogate the contribution of myeloid cells, the most abundant cell type in the mammalian bone marrow, in a clinically relevant mouse model of neutropenia. Long-term genetic depletion of neutrophils and eosinophils results in activation of multipotent progenitors but preservation of HSCs. Depletion of myeloid cells abrogates HSC expansion, loss of serial repopulation and lymphoid reconstitution capacity and remodeling of HSC niches, features previously associated with hematopoietic aging. This is associated with mitigation of interferon signaling in both HSCs and their niches via reduction of NK cell number and activation. These data implicate myeloid cells in the functional decline of hematopoiesis, associated with activation of interferon signaling via a putative neutrophil-NK cell axis. Innate immunity may thus come at the cost of system deterioration through enhanced chronic inflammatory signaling to stem cells and their niches.

## Abstract (web summary)

Innate and adaptive immune cells function in the homeostasis of haematopoietic stem cells (HSC). Here the authors show that myeloid cells are able to reduce the function of HSCs via interferon signaling through a neutrophil-NK cell dependent process.

## Introduction

Hematopoietic stem cell (HSC) pools are positioned at the hematopoietic hierarchical apex and sustain multi-lineage hematopoiesis throughout the mammalian lifetime. They can do so by maintaining relative quiescence, self-renewal, and infrequent divisions during steady-state hematopoiesis. These critical processes are governed by ancillary cells in so-called stem cell niches, which include endothelial and mesenchymal cells in the mammalian hematopoietic system. Recent findings implicate resident innate and adaptive immune cells in the homeostatic regulation of stem cells^1^. In particular, macrophages^2^ and regulatory T cells^3^ are established regulators of hematopoietic stem cells under homeostatic conditions.

The contribution of neutrophils, the most abundant innate immune cell in the human bone marrow, to homeostatic stem cell regulation, however, has remained largely elusive. This is mainly due to the lack of models fulfilling the experimental paradigm for defining HSC-regulating cells, which is (long-term) specific depletion of a candidate regulatory cell, followed by rigorous examination of HSC function. Existing models of neutropenia either induce transient, short-term reduction of neutrophil levels (e.g. by diphtheria toxin deletion systems or antibodies targeting neutrophils) and/or employ genetic strategies that target HSCs themselves, precluding conclusions on the effect of neutropenia on long-term HSC biology^4^.

Granulocytes have been shown, by delivering TNF to endothelial cells, to promote blood vessel growth and hematopoietic recovery in the setting of injury, demonstrated through short-term transfer or depletion of granulocytes following transplantation^5^. Elegant experiments exploiting short-term (2-day) depletion of circulating neutrophils by injection of anti-Ly6G antibody demonstrated modulation of the HSC niche and enhanced HSC retention in the bone marrow through expansion of CXCL12-expressing stromal niche cells^6^. Injection of anti-Ly6G antibody did, however, not lead to reduced neutrophils in the bone marrow and the long-term function of bone marrow-resident HSCs was not tested in these experiments, leaving open the question of neutrophil contributions to HSC physiology over a mammalian lifetime.

Long-term experimental depletion of neutrophils (neutropenia) is not only of conceptual relevance to define their contribution to HSC biology but also of significant clinical relevance, as chronic neutropenia is a common hematological condition associated with leukemic transformation in congenital neutropenia syndromes such as Shwachman–Diamond syndrome (SDS), severe congenital neutropenia (SCN)^7^ and myelodysplastic syndromes (MDS)^8,9^. It remains to be established whether neutropenia in itself contributes to bone marrow failure and malignant transformation in these disorders, perhaps through the induction of replicative stress, DNA damage, and HSC exhaustion. Upon stress factors such as bleeding, infection, or inflammation, the hematopoietic system is capable of rapid adaptation and increasing the production of differentiated hematopoietic cells. This demand-driven hematopoiesis, however, activates quiescent HSCs^10^ thereby attenuating stem cell genomic integrity and function^11–13^. HSCs can be induced out of quiescence by a range of stimuli such as LPS^14–16^, interferon (IFN)-α^17^, IFN-γ^18^, granulocyte colony-stimulating factor (G-CSF)^19,20^, thrombopoietin (TPO)^21^ or IL-1^22^, provoking DNA damage in long-term (LT)-HSCs in vivo, ultimately resulting in stem cell exhaustion.

Here, utilizing a mouse model of profound, sustained, and specific depletion of mature myeloid cells (neutrophils and eosinophils), we demonstrate that HSC integrity and function are conserved, implicating divergent responses of stem and progenitor cells to compensate for myeloid lineage shortages. Unexpectedly, the depletion of myeloid cells attenuated inflammatory signaling in stem cells and their niches via the reduction of NK cell numbers and activation status and abrogated the loss of HSC function in serial transplantation, identifying a neutrophil–NK cell axis as a critical determinant of the functional decline of the hematopoietic system.

## Results

### A mouse model of profound and sustained neutropenia with genetically intact HSCs

To interrogate HSC responses to neutropenia, we exploited our previously reported mouse model of neutropenia, through targeted downregulation of *Sbds* (loss-of-function, the causative event in SDS) in hematopoietic progenitor cells (HPCs) expressing the myeloid transcription factor CCAAT/enhancer binding protein α (C/EBPα, encoded by the *Cebpa* gene)^23^. Neutropenia in this model is caused by a maturation block, specifically at the myelocyte stage of myeloid development^23^, recapitulating the neutropenia caused by a maturation block in (pro)myelocytes observed in human SCN^24,25^ and a subset of SDS patients^26,27^.

**Fig. 1:**
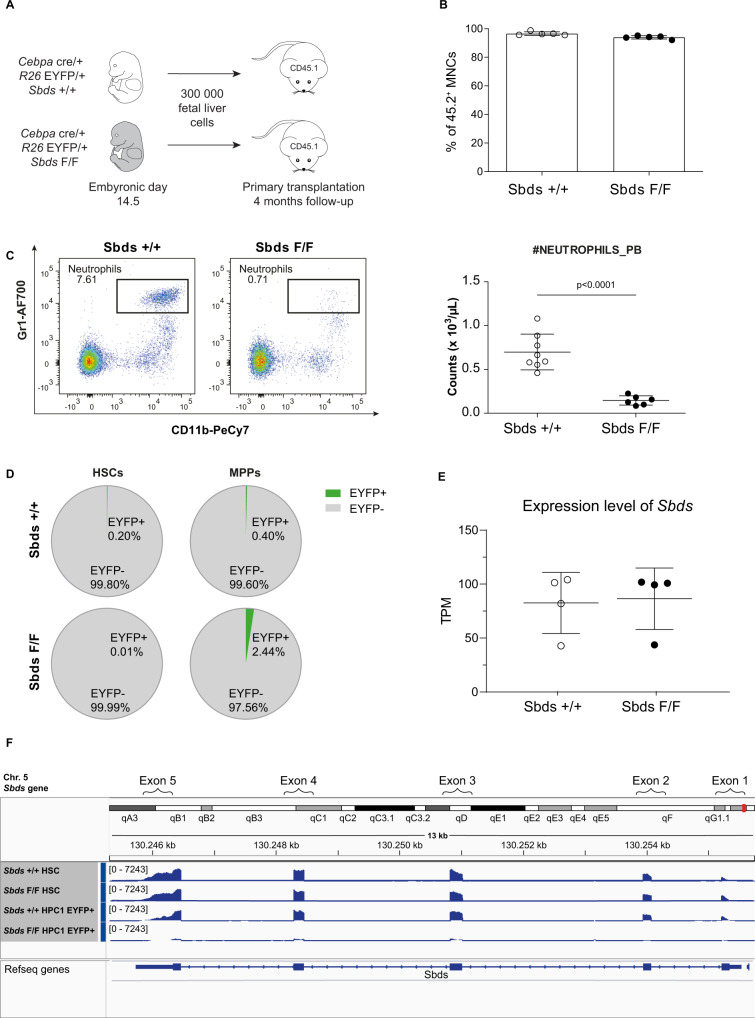
A mouse model of profound and sustained neutropenia with genetically intact HSCs. **A** Schematic illustration of the experimental design for primary transplantation. Data of 4 months post-transplant are presented. **B** Peripheral blood chimerism following primary transplantation. Frequencies of CD45.2 (donor) cells are plotted (*n* = 5). **C** Representative flow cytometry plots of neutrophil (Gr1^+^/CD11b^+^) frequencies and absolute neutrophil counts in PB (*n* = 8^+/+^,6 ^F/F^). **D** HSCs (LKS-Slam) are *Sbds* proficient in the model as indicated by the mean absence of EYFP^+^ cells (as a measure of *Cebpa*-mediated recombination). **E** Unaltered gene expression levels of *Sbds* in HSCs (LKS-Slam) (*n* = 4). **F** Intact exon expression of the *Sbds* gene in LKS-Slam from *Sbds*^F/F^ mice; *Sbds* exon expression in HPC1 (EYFP^−^ and EYFP^+^) from *Sbds*^F/F^ recipients are displayed as references. Data are mean ± SD. A two-sided unpaired *t*-test was performed for statistical analysis. TPM transcripts per kilobase million. Source data are provided as a Source Data file.

Transplantation of embryonic day (E) 14.5 fetal liver cells isolated from *Cebpa*^cre/+^ *R26*^*EYFP/+*^ *Sbds*^F/F^ or *Cebpa*^cre/+^ *R26*^*EYFP/+*^ *Sbds*^+/+^ embryos (CD45.2) into wild-type CD45.1 recipients enables the tracing of *Cebpa Sbds*-depleted or *Sbds*-proficient cells and their progeny based on enhanced yellow fluorescent protein (EYFP) expression. Recipients of the *Cebpa*^cre/+^ *R26*^*EYFP/+*^ *Sbds*^F/F^ fetal liver cells (hereafter *Sbds*^F/F^ or mutants) developed profound neutropenia with an absolute neutrophil count (ANC) of 0.15 ± 0.05 × 10^3^/µL vs. 0.70 ± 0.20 × 10^3^/µL in recipients of the *Cebpa*^cre/+^ *R26*^*EYFP/+*^ *Sbds*^+/+^ fetal liver cells (hereafter *Sbds*^+/+^ or controls), which was stably sustained until sacrificing the animals at 4 months post-transplant (Fig. 1A–C), confirming our previous findings^23^. ANC was also profoundly reduced in bone marrow (2.87 ± 0.93 × 10^5^ vs. 4.4 ± 2.8 × 10^3^ cells/femur/gram body weight, respectively *Sbds*^+/+^ vs. *Sbds*^F/F^) as were eosinophil counts, while red blood cells and platelets were not reduced in mutant recipients. The number of macrophages, previously implicated in HSC regulation^2^ was not altered in mutant mice (Supplementary Fig. 1A–D). Neutropenia was maintained upon serial transplantation into secondary and tertiary recipients, as discussed hereunder (see the section “Preservation of stem cell function and niches in neutropenia”).

Importantly, the HSCs in this model are *Sbds* proficient as indicated by the lack of EYFP^+^ cells within the HSC (LKS CD150^+^CD48^−^ or LKS-Slam) population (Fig. 1D and Supplementary Fig. 1F) and normal levels of *Sbds* transcripts (Fig. 1E, F) (including expression of exon 2, flanked by *LoxP* sites) as compared to reduced levels of expression as a known consequence of *Sbds* recombination in EYFP^+^ HPC1 cells (Fig. 1F)^23^.

Collectively, these data indicate that the mouse model represents a unique system of profound and sustained neutropenia, both in the BM and blood, allowing the study of HSC biology in this condition within the relevant context of human disease (congenital neutropenia with a predisposition for the development of acute myeloid leukemia)^28,29^.

### HSC quiescence is preserved in neutropenia

**Fig. 2:**
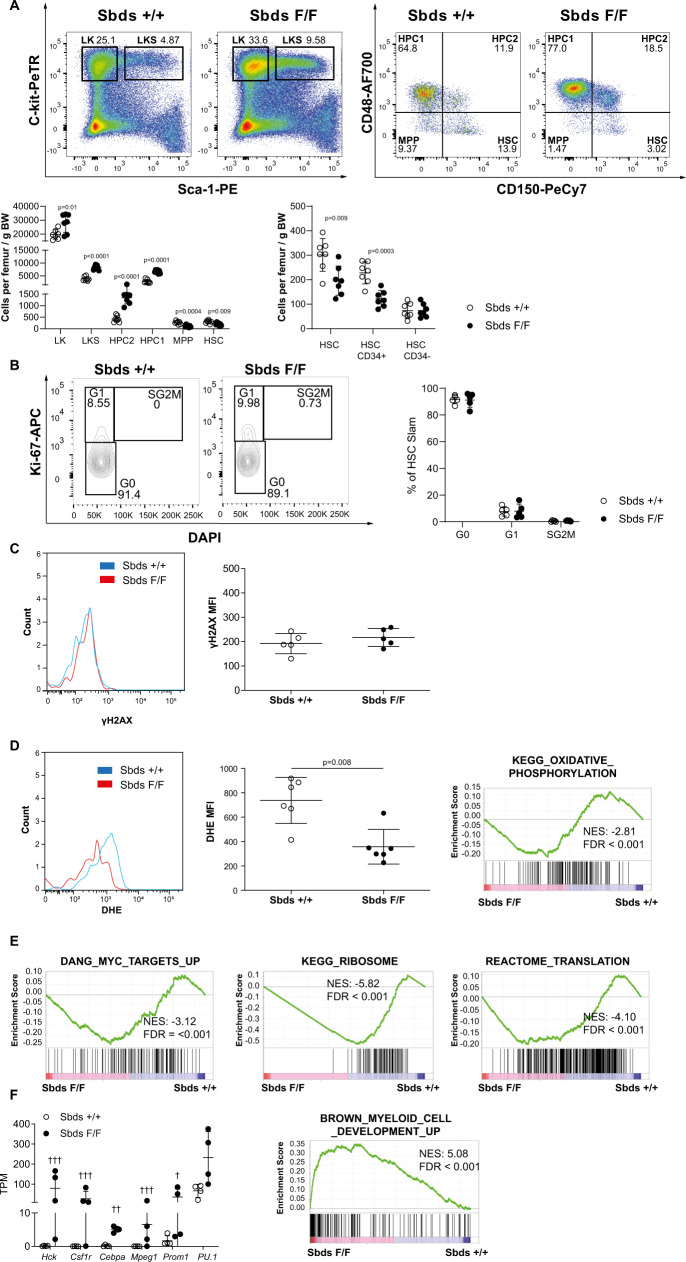
The HSC population in neutropenia displays myeloid priming while remaining quiescent. **A** Representative flow cytometry plots of HSPC frequencies and absolute numbers at 4 months following primary transplantation (*n* = 7). **B** Cell cycle status of HSCs (LKS-Slam) (*n* = 5). **C** Unaltered DNA damage response in HSCs (LKS-Slam) as measured by the accumulation of γH2AX. Median fluorescent frequency (MFI) is plotted (*n* = 5). **D** Reduced mitochondrial ROS levels in HSCs (LKS-Slam) as measured by dehydroethidium (DHE) and transcriptional signature indicative of reduced oxidative phosphorylation (*n* = 6). **B**–**D** Average number of HSCs analyzed is 397 (range 70–841). **E** Transcriptional landscapes indicative of reduced Myc signaling, ribosome biogenesis, and translation in LKS-Slam. **F** Myeloid priming of HSCs (LKS-Slam) as indicated by increased expression of key myeloid commitment genes and enrichment of myeloid differentiation transcriptional programs by GSEA (*n* = 4). Data are mean ± SD. Two-sided unpaired *t*-test was performed for statistical analysis. TPM transcripts per kilobase million. †††FDR < 0.001, ††FDR < 0.01, †FDR < 0.05. NES normalized *p*-value and FDR value of each gene set are listed. GSEA gene sets enrichment analysis, NES normalized enrichment score, FDR false discovery rate. Source data are provided as a Source Data file.

Stress stimuli can induce a cell cycle entry response and provoke DNA damage in LT-HSCs in vivo, ultimately resulting in stem cell exhaustion^11,13,19^. We first asked whether similar cellular events would occur in HSCs in neutropenia. The number of dormant HSCs (LKS-Slam, CD34^−^)^30^, however, was maintained in neutropenia (Fig. 2A). HSCs in neutropenia were predominantly quiescent (in the G0 phase of the cell cycle), comparable to their control counterparts (Fig. 2B). Moreover, DNA damage (responses), associated with replicative stress, were indiscernible between HSCs in neutropenia and controls, as assessed by the accumulation of Ser139-phosphorylated H2AX histone (γH2AX), which forms at the sites of DNA damage (Fig. 2C).

Mitochondrial-derived reactive oxygen species (ROS), thought to act as key DNA-damaging agents in the context of replicative stress^19^ were, unexpectedly, significantly decreased in HSCs in neutropenia as shown by dihydroethidium (DHE) staining (Fig. 2D). Reduced mitochondrial superoxide radicals may signal glycolysis, a metabolic feature associated with stem cell quiescence^31^. In line with this notion, massive parallel RNA sequencing of the HSC (LKS-Slam) population demonstrated enrichment of transcriptional signatures by Gene Set Enrichment Analysis (GSEA) indicative of reduced oxidative phosphorylation and glycolysis in HSCs in the context of neutropenia compared to HSCs isolated from control, non-neutropenic mice (Supplementary Data 1, Fig. 2D). Notably, other key transcriptional programs associated with stem cell activation such as MYC signaling^32^ and protein synthesis/translation^33^ were suppressed in HSCs in the context of neutropenia (Fig. 2E, Supplementary Data 1).

In contrast, multipotent progenitors (MPPs) displayed significantly increased cycling and the number of immunophenotypic MPPs^34^ was significantly decreased in neutropenia in comparison to their counterparts in controls (Fig. 2A and Supplementary Fig. 2A). Increased cycling was associated with the activation of transcriptional programs associated with cell cycle, translation, oxidative phosphorylation, myeloid differentiation, MYC activation and DNA repair in this population in comparison to MPPs from control mice (Supplementary Fig. 2A–C, Supplementary Data 1).

Contributions of the HSC compartment to this actively cycling progenitor pool were suggested by the reduced numbers of committed CD34^+^ HSCs (Fig. 2A) in combination with a striking abundance of transcriptional signatures indicative of myeloid priming of the HSC population, as demonstrated by differential gene expression analysis and GSEA (Fig. 2F). This included significant upregulation of transcripts that encode proteins that are instrumental in myeloid commitment and differentiation such as *PU.1 (*Spi*1)*^35^, *Hck*^36^, *Csf1r*^37^, *Cebpa*^38^, *Mpeg1*^39^ and *Prom1* (encoding CD133)^40^. Note that, although levels of *Cebpa* mRNA were significantly upregulated in the HSC population in neutropenia, they were substantially lower than in downstream progenitor subsets (TPM 5.10 ± 0.85 in HSC vs. 10.34 ± 1.16 in HPC1), and insufficient to drive recombination of the *Sbds* gene (see Fig. 1D–F).

The myeloid commitment of immunophenotypic HSCs in the G0 cell cycle phase seems consistent with a recent report showing that HSCs are able to differentiate into restricted progenitors without undergoing cell division and even before entering the S-phase of the cell cycle^41^ and places this observation in the pathophysiologic relevant context of neutropenia.

Collectively, the data are congruent with the notion of divergent responses of HSC and transit-amplifying progenitor cells to the stress invoked by chronic neutropenia. HSCs may be actively conserved in an inactivated, quiescent, yet myeloid-primed state, with a CD34^+^ subset differentiating and contributing to their downstream early myeloid-biased progeny (MPPs) which is activated and proliferates to expand progenitor cell (HPC) pools to replenish the shortage of neutrophils (Supplementary Fig. 2D).

### Preservation of stem cell function and niches in neutropenia

**Fig. 3:**
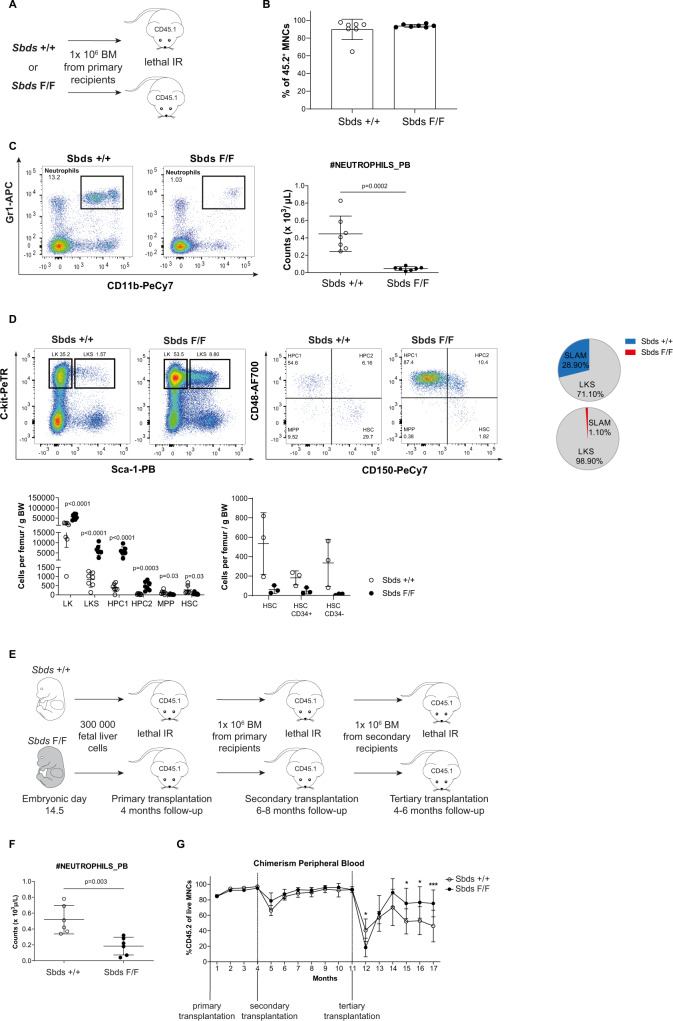
HSC expansion and loss-of-function are abrogated in neutropenia. **A** Schematic representation of the experimental design for secondary transplantation. Data are presented for 6–8 months post-transplant. **B** Peripheral blood chimerism of CD45.2 donor cells (*n* = 7). **C** Neutropenia in secondary recipients. Representative flow cytometry plots of neutrophil (Gr1^+^/CD11b^+^) frequency and absolute neutrophil count (*n* = 7). **D** Representative flow cytometry plots of HSPC frequencies (*n* = 7), the relative contribution of HSC-Slam population within LKS (*n* = 3), absolute numbers of HSPCs, and absolute numbers of CD34^−^ LKS-Slam cells in secondary transplants. Note: The number of CD34^−^ HSCs has expanded in comparison to the first transplant (see Fig. 2a) in controls but not neutropenic mice. **E** Schematic representation of tertiary transplant. **F** Neutropenia 6 months after tertiary transplantation. **G** Enhanced long-term serial repopulation ability of HSCs exposed to sustained neutropenia (*n* = 6). Chimerism as assessed by monthly peripheral blood analysis during serial transplantations is shown (*n* = 5–6). Data are mean ± S.D. **A**–**F** Two-sided unpaired *t*-test was performed for statistical analysis. **G** One-way analysis of variance (ANOVA) with Bonferroni correction was used for multiple comparisons; ***p < 0.001, **p < 0.01, *p < 0.05. Source data are provided as a Source Data file.

To test the functional integrity of HSCs in neutropenia, we next performed serial re-transplantation assays to probe their serial reconstitution ability (Fig. 3). HSCs from neutropenic mice were transplanted serially into secondary and tertiary, lethally irradiated, wild-type CD45.1 recipients. Notably, neutropenia persisted in secondary and tertiary transplants (Fig. 3C, F), as expected, allowing us to test the effect of neutrophil depletion on HSC biology and the hematopoietic system for a total duration of ~17 months.

HSCs from neutropenic mice readily repopulated secondary recipients with almost complete chimerism and reconstituted blood counts to normal levels (Fig. 3A, B and Supplementary Fig. 3A). Preservation of HSC repopulation capacity in neutropenia was confirmed by competitive transplantation of highly FACS-purified HSCs (Supplementary Fig. 4A, B). Of note, the rescue of neutropenia in this competitive setting (Supplementary Fig. 4C) resulted in the normalization of CD45.2 HSPC frequencies (Supplementary Fig. 4D), confirming that altered frequencies in *Cebpa*^*cre/+*^ *Sbds*^F/F^ mice (Fig. 3D) are the result of neutropenia and not HSPC intrinsic alterations. Relative and absolute expansion of the number of (dormant) HSCs was observed in non-neutropenic control mice 6–8 months after secondary transplant (Fig. 3D), congruent with a well-documented phenotype of HSC expansion upon aging^42^. In contrast, this expansion of immunophenotypic HSCs was not observed in neutropenia (Fig. 3D).

Strikingly, when equal numbers of mononuclear bone marrow cells of secondary transplant recipients were transplanted into tertiary recipients, this non-expanded HSC pool outperformed control HSCs from non-neutropenic mice in their multi-lineage reconstitution ability (Fig. 3E, G and Supplementary Fig. 3B, C), indicative of increased/preserved long-term self-renewal capacity of HSCs in the setting of neutropenia. In particular, the contribution of transplanted HSCs from neutropenic mice to lymphopoiesis significantly exceeded that of HSCs from non-neutropenic mice (Supplementary Fig. 3C). Taken together, control HSCs expand upon transplantation, a feature which, in concert with the loss of serial repopulation ability in tertiary transplants and impaired contribution to the lymphoid lineage, is reminiscent of stem cell aging^42^, while these events are abrogated in the context of neutropenia.

Findings were corroborated in an independent neutropenia cohort with long-term follow-up (12 months after initial transplant of *Cebpa*^*cre/+*^ *Sbds*^F/F^ cells (minimizing potential effects of transplantation stress on the outcome) (Supplementary Fig. 5A–C) followed by competitive transplants to test HSC function (Supplementary Fig. 5D–H). The number of immunophenotypic HSCs after 12 months was significantly lower in neutropenia in comparison to controls (Supplementary Fig. 5C), yet these remaining HSCs outcompeted wild-type immunophenotypic HSCs (CD45.1) in a competitive transplant setting in which CD45.2 HSCs from neutropenic mice constituted only 7.7% (±11.17) of the HSCs pool (Supplementary Fig. 5F) but reconstituted >60% of multi-lineage hematopoiesis (Supplementary Fig. 5G, H).

Exhaustion or malignant transformation was not observed upon serial transplantation of bone marrow from neutropenic mice. Prospective isolation of highly purified mesenchymal (niche-CD31^−^CD51^+^Sca1^+^) and endothelial (niche-CD31^+^Sca1^+^) cells (Supplementary Fig. 6A) showed an increased frequency of these endosteal niche cells in neutropenia (Supplementary Fig. 6B, C), consistent with a previous report of niche expansion upon short-term neutrophil depletion^6^. Niche preservation was associated with transcriptional programs indicating cell cycle regulation in both mesenchymal and endothelial niches (Supplementary Fig. 6B, C), suggesting that maintenance of proliferation may be implicated in the preservation of these niches.

### Neutropenia results in the abrogation of interferon signaling in HSCs and their niches

The hematopoietic features mitigated by the depletion of neutrophils (HSC expansion and loss of function as well as reduction of stem cell niches at the endosteal surface) have previously been associated with aging^42–44^ although it should be emphasized that our experimental model of serial transplantation does not formally represent a model of natural aging.

**Fig. 4:**
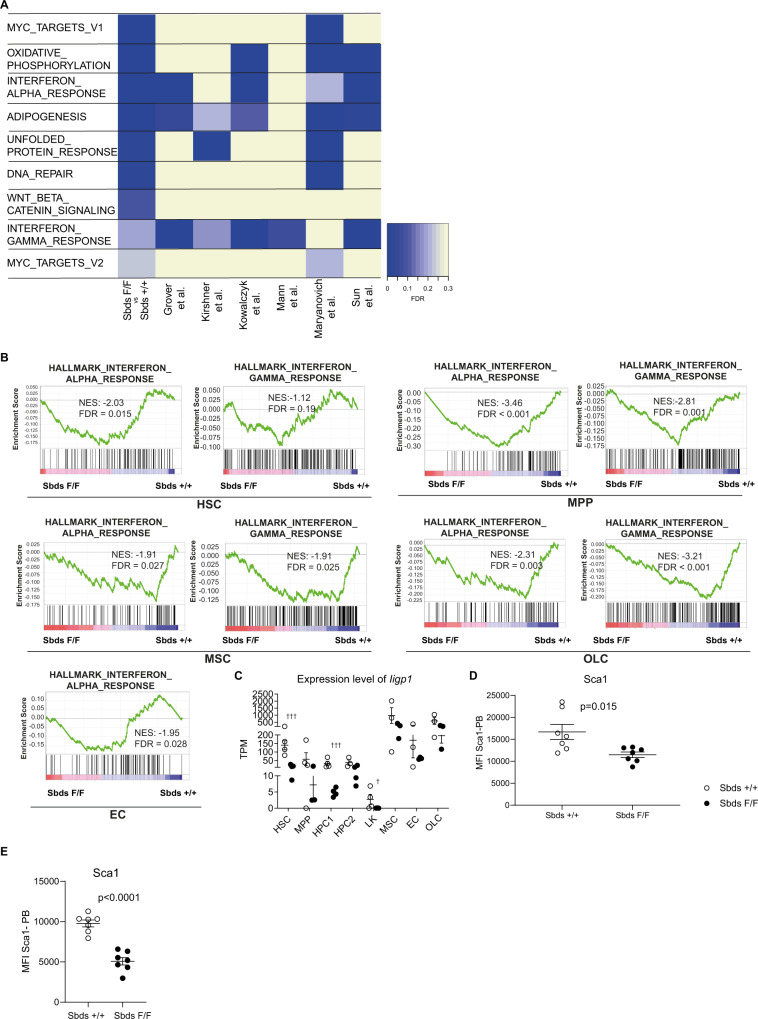
Interferon signaling is abrogated in hematopoietic and niche cells in neutropenia. **A** All hallmark gene sets significantly enriched in HSCs from non-neutropenic (Sbds^+/+^) mice in comparison to neutropenic (Sbds^F/F^) mice are listed. Data from reported studies of (sc)RNAseq in HSCs (LKS-slam) was re-analyzed by GSEA (Hallmark). Activation of interferon signaling (either interferon alpha and/or interferon-gamma) is identified as a transcriptional signature of aged HSCs in all reported studies and is abrogated in neutropenic mice. **B** Abrogation of transcriptional IFN signaling in both HSPCs and mesenchymal/endothelial niche cells from the bone marrow of neutropenic mice (4 months after primary transplantation) (*n* = 3–4). **C** Reduced expression of the canonical IFN transcriptional target interferon-inducible GTPase1 (*Iigp1)* in both HSPCs and niche cells isolated from neutropenic mice (4 months after primary transplantation) (*n* = 3–4). **D** Reduced expression of Sca1 in HSCs from neutropenic mice (*n* = 7). **E** Persistent reduction of Sca1 expression of HSC-Slam in secondary transplantation (after 8 months follow-up) (*n* = 7). Data are mean ± SEM. **D** and **E**: Two-sided unpaired was performed for statistical analysis. **C**: ††† FDR < 0.001, †† FDR < 0.01, † FDR < 0.05. TPM transcripts per kilobase million. Source data are provided as a Source Data file.

To explore the underlying molecular mechanisms governing long-term HSC quiescence and functional conservation of the hematopoietic system in neutropenia, and focus on those that may be relevant for natural aging, we compared genes and transcriptional programs differentially expressed in HSCs in neutropenic mice vs. non-neutropenic controls (Fig. 4A) to those reported as differentially expressed in young vs. old HSCs in literature (Fig. 4A, methods). Unbiased transcriptional program re-analysis of all reported RNAseq datasets on aging HSCs (LKS-Slam) identified transcriptional signatures indicating activation of interferon (IFN) signaling to be common to all reported datasets and HSCs in non-neutropenic vs. neutropenic mice (Fig. 4A, B).

Expression of *Iigp1*, the gene encoding interferon-inducible GTPase 1 (also known as *Iigp*, *Irga6,* or *Ifgga1*), was significantly reduced in HSCs in neutropenia (11-fold, *p*-adj = 2.5 × 10^−5^; Fig. 4C). Interferon-inducible GTPase 1 is a key regulator of cellular response to interferons (IFN)^45^ as well as the single most differentially upregulated (approximately 10-fold) gene in LKS-Slam HSCs in response to IFNα^17^. Furthermore, support for the activation of IFN signaling is provided by the upregulation of Sca1 in HSCs from primary and secondary transplant non-neutropenic mice (Fig. 4D, E), a known consequence of induction by interferons^17,46^. Notably, the rescue of neutropenia in the competitive transplant setting (Supplementary Fig. 4A) resulted in the normalization of Sca1 and *Iigp1* levels (Supplementary Fig. 4E), confirming that reduction in IFN signaling was a consequence of neutropenia and not HSPC intrinsic alterations.

IFN signaling is arguably the best-documented driver of HSC activation and exhaustion upon chronic exposure in mammals^47^. Quiescent HSCs are activated by IFNγ in response to chronic infection^18^ and IFNα activates dormant HSCs^17,48^ and impairs HSC self-renewal^46^. Importantly, activation of IFN signaling has also been recognized as a driver of physiologic HSC exhaustion in mice^48^, but the cellular sources and determinants of this tonic IFN signaling have remained elusive.

Notably, upregulation of *Iigp1* and transcriptional activation of IFN signaling is not only a prominent feature of aging HSCs but also of their aging mesenchymal niches^49^. Moreover, IFN signaling in mesenchymal niche cells has been reported to negatively affect their maintenance and hematopoietic support capacity^50^. Strikingly in this context, mesenchymal stromal cells (MSCs), osteolineage cells (OLCs), and endothelial cells in neutropenia, like HSCs, displayed reduced expression of *Iigp1* and the transcriptional signatures indicative of activated IFN signaling (Fig. 4B, C, Supplementary Data 1).

### Natural killer (NK) cells induce interferon signaling in HSPCs and are reduced in neutropenia

**Fig. 5:**
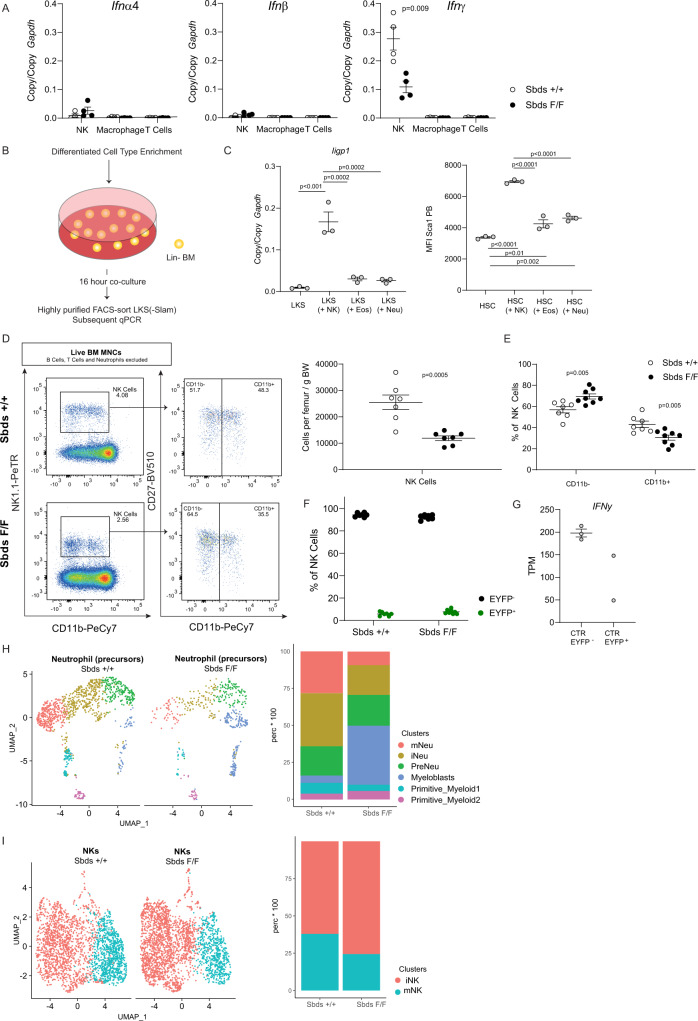
NK cells are the main source of interferons in the bone marrow, induce interferon signaling in HSPC, and are deficient in neutropenic mice. **A** Relative expression of *interferon alpha4* (*Ifnα4*), *interferon beta* (*Ifnβ*), and *interferon gamma* (*Ifnγ*) (by qPCR) of distinct cell types isolated from *Sbds*^F/F^ and *Sbds*^+/+^ bone marrow (*n* = 4). **B** Experimental design for in vitro co-culture of hematopoietic progenitors and various differentiated cell types. Bone marrow or spleen was enriched for differentiated cell types after which it was co-cultured with lineage-negative HSPCs for 16 h. **C** Activation of interferon signaling in HSPCs by NK cells as demonstrated by induction of expression of *Iigp1* and protein expression of Sca1 (*n* = 3). **D**–**G** Reduced NK cell numbers and impaired maturation status in neutropenia. **D** Reduction of NK cells (EYFP^−^) in neutropenic bone marrow. Representative flow cytometry plots of NK (NK1.1^+^) cell subsets are shown (*n* = 7). **E** Maturation status of EYFP^−^ NK cell population as indicated by fraction of CD11b^+^ cells. **F** The vast majority of NK cells is EYFP^−^ (**E** and **F** *n* = 7^+/+^,8^F/F^). **G** EYFP+ subset of NK cells did not differentially express *Ifnγ* (*n* = 3^+/+^,2^F/F^). **H**, **I** Delineation of the myeloid lineage and NK cell subsets by scRNAseq performed 12 months after transplantation of *Cebpa-cre Sbds*^+/+^ or ^F/F^ cells, confirming the relative depletion of mature neutrophils and the subsetting of the NK cell cluster into an immature (iNK) and a mature (mNK) fraction (**H** and **I** *n* = 2). Data are mean ± SEM. TPM transcripts per kilobase million. Two-sided unpaired *t*-test or one-way ANOVA was performed for statistical analysis. Source data are provided as a Source Data file.

Next, we examined the cellular determinants of activated IFN signaling in the bone marrow. We were unable to detect noticeable levels of IFN transcripts (*Ifnα4*, *Ifnβ*, *Ifnγ*) in hematopoietic cells throughout the myeloid lineage, including mature neutrophils isolated from mouse bone marrow (Supplementary Fig. 7A). Mapping of IFN gene expression in other marrow cells, including niche subsets, T-cells, macrophages, eosinophils and NK cells using qPCR (Fig. 5A and Supplementary Fig. 7A, B), demonstrated that *Ifnγ* expression in (NK1.1^+^) NK cells exceeded the expression of *Ifnα4* and *Ifnβ* transcripts (>30-fold) and the expression of this gene in any other examined bone marrow cell type by >100-fold (e.g. 0.3 ± 0.16 copies/copy GAPDH in NK cells vs. 0.002 ± 0.001. in CD3^+^ T-cells, a cell type previously shown to express IFN in the bone marrow^51^), identifying NK cells as the likely dominant source of interferons, and in particular IFNγ, in the steady-state bone marrow.

In line with this, co-culture of HSPCs with NK cells, not neutrophils or eosinophils, resulted in consistent and robust activation of IFN signaling (*Iigp1* expression and increased protein expression of Sca1 as a well-known consequence of IFN signaling)^17,46^ (Fig. 5B, C) and transient NK cell depletion by anti-NK1.1 antibody administration for 10 weeks mitigated transcriptional IFN signaling in HSCs (Supplementary Fig. 7D–G), although the reduction in expression of *Sca1* and *Iigp1* did not reach statistical significance in this experimental setting (Supplementary Fig. 7G). Together, these data identify NK cell-derived interferons as a key determinant of steady-state interferon tonus in the bone marrow. Consistent with this, reduced IFN signaling in HSCs and their niches in neutropenic mice was associated with strikingly reduced NK cell numbers in the bone marrow (Fig. 5D), reminiscent of a previous report showing that neutrophils are essential for NK cell maturation, activation, and function in the spleen^52^. In particular, the differentiated CD11b^+^ subset of NK cells, previously associated with superior ability to secrete cytokines and superior effector function^53,54^, was reduced in neutropenia (Fig. 5D, E). Rescue of neutropenia in the competitive transplant setting (Supplementary Fig. 4A) equilibrated the skewing towards less activated NK cells (Supplementary Fig. 4F), confirming that altered frequencies in *Cebpa*^*cre/+*^ *Sbds*^F/F^ mice (Fig. 5E) are the result of neutropenia and not NK intrinsic alterations. Genetic targeting of *Sbds* in NK cells was excluded by demonstrating that the vast majority of NK cells did not express EYFP and EYFP^−^ NK cells were *Sbds* proficient (Fig. 5F and Supplementary Fig. 7C). Furthermore the small EYFP^+^ subset of NK cells did not differentially express *Ifng* (Fig. 5G).

### Neutrophil–NK cell interaction analyses identify CCL6–CCR2 signaling as a candidate determinant of NK cell number and activation

To delineate the potential molecular mechanisms underlying the communication between neutrophils and NK cells, we performed scRNAseq of the neutropenic bone marrow (12 months after transplantation of *Cebpa*^*cre/+*^ *Sbds* ^+/+^ or ^F/F^ cells). To this end, bone marrow cells were subsorted into HSC + MPP (LKS CD48^−^), HPC1 (LKS CD48^+^CD150^−^), B and T cells (B220^+^, CD3^+^), NK cells (NK1.1^+^ NKp46^+^) and a myeloid rest fraction (B220^−^, CD3^−^, NKp46^−^, and NK1.1^−^) and sorted fractions pooled together to obtain a robust representation of all bone marrow cell types in the scRNAseq data (Supplementary Fig. 8A, B). Cell clusters were annotated by assessing canonical cell type markers as described in the “Methods” section. Transcriptional analysis of the HSC and MPP fraction (Supplementary Fig. 8C) confirmed *Sbds* proficiency (Supplementary Fig. 8D), transcriptional myeloid priming (Supplementary Fig. 8E), and increased expression of *Iigp1* (Supplementary Fig. 8F). Delineation of the myeloid lineage confirmed the depletion of mature neutrophils with a relative increase in myeloid progenitors/myeloblasts (Fig. 5H and Supplementary Fig. 9A). NK cells could be subsetted in an immature and mature/activated subsets (based on the expression of *Il7r*, *Prf1,* and *Itgam*) with a relatively larger fraction of mature/activated NK cells in control mice (Fig. 5I and Supplementary Fig. 9B) (in line with previous data, Fig. 5E). *Ifny* was predominantly expressed in NK cells (Supplementary Fig. 10A) (in line with qPCR data, Fig. 5A) while expression of *Ifna* and *Ifnb* were not detectable in scRNAseq, in line with the lower expression levels of these genes.

Analysis of the intercellular communication between neutrophils and NK cells based on possible ligand–receptor crosstalk using CellChat, which predicts the major signaling routes and how they integrate into cellular function, using network analysis and pattern recognition approaches^55^ predicted the existence of a CCL6–CCR2 signaling axis in control mice but not in neutropenic mice. Importantly, CCL6 has been established as a chemokine recruiting interferon-gamma-producing NK cells in experimental settings^56^. Expression of *Ccl6* was distinctly higher in neutrophils (precursors) in comparison to any other cell type in the bone marrow (Supplementary Fig. 10C, left panel), with the highest expression in mature neutrophils, which was significantly decreased in these cells in neutropenia (Supplementary Fig. 10E). In line with these findings, CCL6 protein concentration was significantly lower in the bone marrow plasma of neutropenic mice (Supplementary Fig. 10D). Expression of *Ccr2* was notably high in NK cells (Supplementary Fig. 10C, right panel), and in particular in the mature subset (Supplementary Fig. 10F).

Collectively, the data are consistent with the view that neutrophils may induce activation of IFN signaling in HSCs and their niches indirectly, via recruitment and/or expansion of IFN-producing NK cells, thus suggesting the existence of a neutrophil–NK cell axis as a determinant of inflammatory signaling associated functional decline of the hematopoietic system.

## Discussion

Stress hematopoiesis in experimental conditions such as bleeding, infection, or administration of G-CSF may lead to replicative stress, DNA damage, exhaustion, and perhaps the malignant transformation of HSCs^12,57^. How the hematopoietic system responds to demands resulting from a shortage of mature myeloid cells, in particular, neutrophils (a defined single lineage) remains understudied.

Here, in a mouse model recapitulating the neutropenia observed in human preleukemic disease, we demonstrate that HSC integrity and functional capacity remain conserved during sustained neutrophil depletion. HSCs did not display replicative stress, DNA damage, exhaustion, or leukemic transformation. Rather, the HSC population was maintained in a quiescent state, reflected by the preservation of function in serial and competitive transplantation.

The stem cell pool in neutropenia becomes primed for myeloid differentiation without entering the cell cycle, consistent with recent data showing that stem cell differentiation can occur in G0, uncoupled from proliferation^41^. In contrast, MPPs are activated and increase their cell cycle status, consistent with a model in which transit-amplifying cells replenish myeloid progenitor cell pools (perhaps congruent with emerging data that ST-HSC/MPP have substantial self-renewing capacity^58^) with active preservation of a quiescent compartment of long-term repopulating HSCs to secure long-term hematopoiesis.

The data add to the evolving understanding of stem cell heterogeneity and its implication for hematopoiesis. Our data indicate that a long-term supply of myeloid progenitors can occur through myeloid differentiation of a subset of stem cells while sparing a small pool of dormant HSCs with long-term multi-lineage serial repopulation ability.

Initially unanticipated, the data identify neutrophils as critical determinants of the functional decline of the hematopoietic system. Depletion of neutrophils resulted in abrogation of HSC expansion, conservation of long-term repopulating and lymphopoietic ability of HSCs, as well as preservation of their mesenchymal and endothelial niches.

It is of interest that these hematopoietic features mitigated by the depletion of neutrophils are hallmark features of hematopoietic aging, a condition characterized by an increase of myeloid cells in the bone marrow. The neutropenia model employed by us, however, does not represent a formal model of natural aging and future experiments in systems where neutrophils are selectively and profoundly deleted in steady-state hematopoiesis will have to establish whether neutrophils are key drivers of natural aging.

The mechanisms through which myeloid cells drive the deterioration of the hematopoietic system remain largely to be defined. Our data indicate an interesting association with the activation of IFN signaling. Computational analyses of transcriptional networks in aging HSCs demonstrated that activation of IFN signaling is a strikingly common feature between published datasets, which was attenuated in HSCs and their mesenchymal and endothelial niches in neutropenia. Chronic interferon signaling in HSCs and niche cells is a well-documented driver of the functional decline of HSCs and their niches^48,50,59^, but the cellular components driving the activation of IFN signaling in HSCs and their niches have remained uncertain. Our data suggest the existence of a neutrophil–NK cell axis as a player in this chronic inflammatory signaling in the bone marrow. Expression data, demonstrating high expression of *Ifng* in NK cells, with no detectable expression of *Ifna* or *Ifnb*, suggest that type II IFN signaling may be dominant, but contributions of type I interferon signaling to this proposed axis cannot be excluded. Moreover, other mechanisms may be involved in the preservation of HSCs after the depletion of neutrophils. In this context, the physiological significance of the marked downregulation of *Iigp1* and the upregulation of *PU.1*, previously described to regulate HSC proliferation and pool size during inflammatory stress^60^, will be worth exploring in follow-up studies.

The proposed link between neutrophils and NK cells provides (patho)physiological relevance to an earlier report demonstrating that neutrophils are essential for NK cell maturation, activation, and function in the spleen and that NK cell numbers and maturation are attenuated in neutropenic patients^52^. Future studies may also test interferon signaling and HSC function in neutropenic disorders to assess the human disease relevance of our findings.

Mechanisms of neutrophil-induced NK cell proliferation and IFN production remain to be fully elucidated. ScRNAseq of the neutropenic bone marrow predicted cellular crosstalk between mature neutrophils expressing CCL6, a known chemoattractant for interferon-producing NK cells, and activated *Ccr2*-expressing NK cells, a notion that we substantiated by demonstrating reduced bone marrow plasma levels of CCL6 in neutropenia. However, interactions between neutrophils and NK cells are likely to be multifactorial, comprising granule constituents such as arginase, neutrophil elastase, lactoferrin, defensins, and serine proteases (reviewed in ref. 61), and the molecular mechanisms defining the neutrophil–NK cell activation axis remain largely to be elucidated.

We cannot formally exclude the possibility that other, yet unknown, molecular consequences of *Sbds*-deficiency in the myeloid lineage contribute to the observed phenotypes. We have, however, demonstrated *Sbds* proficiency in both HSCs and NK cells in the model and previously characterized, in detail, the cellular and transcriptional alterations in the myeloid lineage upon deletion of *Sbds* from *Cebpa*-expressing cells^23^, showing that this results in specific attenuation of lineage progression in myelocytes (due to apparent SBDS dependency of these cells) while leaving other myeloid progenitors functionally unperturbed. The reported clinical association between neutropenia and NK cell deficits in non-SDS neutropenia patients^52^ further supports the view that HSC effects are related to the loss of neutrophils and NK cell interactions, independent of the *Sbds*-deficient context.

Moreover, we cannot formally exclude a contribution of the depletion of eosinophils in the *Cebpa-cre* model to the rescue of the functional decline of the hematopoietic system and the reduction of NK cell numbers. Future strategies, genetically targeting these myeloid subsets specifically, are anticipated to further delineate the contribution of eosinophils to these events.

The implication of activated interferon signaling in stem cells and their niches as drivers of the functional decline of the hematopoietic system, as supported by the current data, may carry further relevance to the understanding of hematopoietic diseases associated with the functional decline of the system. In particular, it may shed further light on the selective forces driving the clonal hematopoiesis (CH) associated with aging and an increased risk for malignant transformation and cardiovascular disease^62,63^. It has recently been shown that activated IFNγ signaling, in the experimental context of infection or exogenous administration, is a strong driver of the clonal expansion of cells carrying loss-of-function mutations in the gene *DNMT3A*, by far the most prevalent mutation found in CH. The demonstration of activated IFNγ signaling over time in our mouse model, the elucidation of its cellular determinants, and the proof-of-principle that interference with this driving axis (via depletion of neutrophils) attenuates functional decline of the system associated with hallmark features of hematopoietic aging, is anticipated to direct future investigations into the modulation of tonic interferon signaling in the bone marrow and its consequences for the emergence of CH.

## Methods

### Mice and genotyping

All mice were bred and maintained in the Experimental Animal Center of Erasmus Medical Center (EDC) in Rotterdam, and all work was performed in accordance with Dutch legislation and following institutional ethical approval by the Animal Welfare/Ethics Committee of the EDC (IvD and DEC Rotterdam).

*Cebpα*^*cre/+*^ *R26*^*EYFP*/+^ *Sbds*^*F/+*^ mice were generated in-house and were of C57/BL6 background. Genotyping of *Cebpα*^*cre/+*^, *R26*^*EYFP*/+^, and *Sbds*^+/+^ or *Sbds*^F/F^ mice or fetuses were reported previously^23^. Recipients for transplantations were purchased from Charles River (Ly5.1, B6.SJL *Ptprc*^*a*^*Pepc*^*b*^/BoyCrl). All recipient mice were male and female and 8–12 weeks old at the time of transplantation. NK depletion experiments were conducted on 6-month-old C57BL/6J male mice, also purchased from Charles River. Animals were maintained in specific pathogen-free conditions in the EDC. Mice were housed in groups of maximum of five animals (at least 100 cm^2^ per animal) under a standard 12 h light/dark cycle, with access to food and water ad libitum and an ambient temperature of 21–23 °C with air humidity maintained between 40% and 70%. All mice were euthanized by cervical dislocation.

### Collection of peripheral blood/bone marrow/bone fraction samples

The collection of peripheral blood (PB), bone marrow (BM), and bone fraction (BF) cells were performed as previously described^23,64^. In brief, PB was collected by cheek puncture monthly and at the end of follow-up periods. Complete blood count was measured on Scil Vet ABC Plus hematology analyzer (Scil Animal Care). When applicable, plasma was collected by centrifuging an excess amount of PB in plasma separator tubes (365985, BD Microtainer) following the manufacturer’s instructions and snap-frozen in liquid nitrogen. Bones of the lower limbs (Hip, Femur, Ilium) were used for BM and BF isolation. Bones were crushed in 3 × 2 × 1 mL of FACS buffer (PBS + 0.5% FCS) with sterilized mortar and pestle. Where applicable, BM supernatant was collected by flushing 1 femur with 30 G Micro-Fine Insulin Syringe (324870, BD) containing 300 µL sterile PBS. BM cells were pelleted by centrifuging, and BM supernatant was transferred to a clean Eppendorf tube and snap-frozen in liquid nitrogen. BM cell suspension was passed through a 40 µM filter. RBCs in BM were lysed in 2 mL ACK lysing buffer (Lonza) for 4 min on ice and washed in 20 mL FACS buffer. BF cells were prepared by incubating bone fragments after crushing in 2 mL 0.25% Collagenase Type I (07902, STEMCELL Technologies) for 45 min in a 37 °C water bath, with vortexing every 15 min, washed in 20 mL FACS buffer and passed through a 40 µM filter again. All centrifuging steps were performed at 1600 RPM (~500 G) for 5 min at 4 °C (BM/BF) or RT (PB). Mononuclear cells were quantified with Bürker counting chamber (631-0920, Marienfeld) or the Countess II Automated Cell Counter. Excess BM cells were cryopreserved in a freezing medium containing 90% FCS and 10% DMSO.

### Transplantations

Transplantation of fetal liver (FL) and BM cells was performed as previously described^23,64^. In brief, recipient SJL mice were lethally irradiated (8.5 Gy) on a gamma-cell irradiator (Gammacell 40 for primary and secondary transplantations, IBL637 for tertiary and other transplantations) the day before transplantation. For primary transplantations, FL cells were isolated from E14.5 embryo of pregnant female mice bearing *Cebpα*^*cre/+*^ *R26*^*EYFP*/+^ *Sbds*^+/+^ or *Sbds*^F/F^ FL. A piece of fetus tissue (limb) was collected for genotyping purposes. FL was dissociated in PBS by pipetting up and down. Red blood cells (RBCs) from FL were lysed in 500 µL ACK lysing buffer (Lonza) for 4 min at RT and washed in 2 mL PBS. 3 × 10^5^ mononuclear FL cells were intravenously (i.v.) injected per recipient. For secondary and tertiary transplantations, mononuclear BM cells freshly isolated from 2 control mice or 2 mutant mice were pooled, 1 × 10^6^ cells were i.v. injected per recipient. For competitive transplantation, LKS-Slam cells were stained and FACS sorted from mononuclear bone marrow cells coming from primary recipients. Each lethally irradiated SJL recipient mouse was transplanted with 67 LKS-Slam cells and 200,000 mononuclear bone marrow cells from an SJL (45.1) competitor, unless otherwise stated. Cell suspensions were prepared in PBS and passed through 40 µm filter caps prior to transplantation. Each recipient received 200 µL cell suspension.

### In vivo NK depletion

Six-month-old C57BL/6J were intraperitoneally injected three times per week for 10 weeks with 30 μg NK1.1 (clone PK136) depleting antibody or its isotype control. A booster shot of 300 μg was given once every two weeks. The depleting antibody was provided by Polpharma Biologics. The Mouse IgG2a isotype control was purchased at BioXCell (BE0085).

### Flow cytometry

PB was diluted in FACS buffer at 1:10 (10 µL PB + 90 µL FACS buffer) and stained with antibodies against surface markers for 20 min at RT in the dark. Subsequently, RBCs were depleted with 2 mL IOTest 3 lysing solution (A07799, Beckman Coulter) for 10 min at RT. BM and BF cells were stained in FACS buffer for 20 min on ice in the dark. All washing steps were performed by adding 3 mL FACS buffer to the sample and centrifuging at 1600 RPM (~500 G) for 5 min at RT (PB) or 4 °C (BM/BF).

To identify differentiated cells in the PB and BM, the following antibodies were used: Ly-6G/Ly-6C (Gr-1) AF700 RB6-8C5 (BioLegend) (1:100), Ly-6G/Ly-6C (Gr-1)—APC RB6-8CS (BioLegend) (1:100), CD11b PE-Cy7 M1/70 (BioLegend) (1:100), CD11b APC-CY7 M1/70 (BioLegend) (1:100), Ly6G AF700 1A8 (BioLegend) (1:100), Ly6C BV510 HK1.4 (BioLegend) (1:100), CD115 APC AFS98 (BioLegend) (1:100), B220 eFluor450 RA3-6B2 ((eBioscience)) (1:200), B220 PE RA3-6B2 (BioLegend) (1:200), F4/80 PE-Cy7 BM8 (SONY Biotechnology (1:100), CD3e BV510 145-2C11 (BD Bioscience (1:100), CD3 eFlour450 17A2 (eBioscience) (1:100), CD43 PE-Cy7 Ly-48 (BioLegend) (1:100), CD27 BV510 LG.3A10 (BioLegend) (1:100), NK-1.1 PE/Dazzle 594 PK136 (BioLegend) (1:300) and NKp46 eFlour660 29A1.4 (eBioscience) (1:50), CD19 Biotin 6D5 (BioLegend) (1:100), CD11b BB700 (PerCP Cy5.5) M1/70 (BioLegend) (1:100), CD193 PE J073E5 (BioLegend) (1:100), CD170 (Anti-Siglec-F) BV421 S17007L (BioLegend) (1:100), CD49b PE HMα2 (BioLegend) (1:100), CD200R3 PE-Cy7 Ba13 (BioLegend) (1:100) and Anti-FcεRIα APC MAR-1 (BioLegend) (1:100).

To identify HSPCs in the BM, cells were first stained with lineage (Lin) cocktail containing biotinylated antibodies against Ly-6G/Ly-6C (Gr-1) Biotin RB6-8C5 (BD Bioscience) (1:100), CD11b Biotin M1/70 (BD Biosciences) (1:100), Ter119 Biotin TER-119 (BD Biosciences) (1:100), CD3e Biotin 145-2C11 (BD Biosciences) (1:100), CD4 Biotin GK1.5 (BD Biosciences) (1:100), CD8 Biotin 53-6.7 (BD Biosciences) (1:100) and B220 Biotin RA3-6B2 (BD Biosciences) (1:100). After one washing step, cells were incubated with Streptavidin Pacific Orange (Life Technologies) (1:200), together with a combination of the following antibodies: Sca1 Pacific Blue E13-161.7 (BioLegend) (1:100), CD48 AF700 HM48-1 (BioLegend) (1:100), CD150 PE-Cy7 TC15-12F12.2 (BioLegend) (1:100), c-Kit APC 2B8 (BD Biosciences) (1:100), c-Kit PE-CF594 2B8 (BD Biosciences) (1:100), CD16/32 APC-Cy7 2.4G2 (BD Biosciences) (1:100), CD34 eFluor450 RAM34 (eBioscience) (1:50) and CD34 eFluor660 RAM34 (eBioscience) (1:50). To identify distinct niche populations (MSCs and OLCs), BF cell suspension was stained with the following antibodies: Ter119 BV510 TER-119 (BioLegend) (1:100), CD51 PE RMV-7 (BioLegend) (1:100), CD31 PE-CF594 MEC 13.3 (BD Biosciences) (1:100), CD140α APC APA5 (eBioscience) (1:100), CD45.1 Biotin A20 (BioLegend) (1:100), CD45.2 Biotin 104 (BioLegend) (1:100), NK-1.1 Biotin PK136 (BioLegend) (1:100), CD45.1 APC-Cy7 A20 (BioLegend) (1:100) and CD45.2 APC-Cy7 104 (BioLegend) (1:100). To identify chimerism and distinguish donor cells from host cells, we used CD45.2 APC-Cy7 or APC 104 (BioLegend) (1:100) and CD45.1 PE A20 (BioLegend) (1:100). Mononuclear cell gating and doublet exclusion (Supplementary Fig. 1A) was always preceded by dead cell exclusion which was performed using 7-AAD BB700 (PerCP Cy5.5) (BioLegend) (1:100), Zombie NIR Fixable Viability Kit APC-Cy7 (BioLegend) (1:800) or LIVE/DEAD Fixable Aqua Dead Cell Stain Kit (Invitrogen) (1:100). All FACS analysis were performed on BD LSR II flow cytometer (BD Biosciences) and data were processed with FlowJo software (Version 7.6.5, Tree Star). All cell sorting experiments were performed on the BD FACSAria III flow cytometer (BD Biosciences).

### Cellular stress and cell cycle assays

Cellular stress and cell cycle assays were performed as previously described^64^. In brief, after surface antibody staining, ~1 × 10^6^ BM cells were used for the assays. For the cell cycle and γH2AX analysis, cells were fixed, permeabilized, stained, and washed using Fixation/Permeabilization Solution Kit (554714, BD Biosciences) following the manufacturer’s instructions. Cell cycle analysis was performed using anti-Ki-67 Set (556027, B56, BD Biosciences); γH2AX levels were detected using anti-γH2AX (560447, N1-431, BD Biosciences), according to the manufacturer’s recommendations. For oxidative stress analysis, cells were resuspended in 200 µL HBSS buffer containing 1 µM dihydroethidium (D7008, Sigma-Aldrich) and incubated for 30 min at 37 °C. All assays were analyzed on BD LSR II flow cytometer (BD Biosciences).

### Co-culture of progenitors

Wild-type BM was lineage depleted by magnetic-activated cell sorting (MACS) according to the manufacturer’s protocol (130-090-858, Miltenyi Biotec). Neutrophil, NK, and eosinophil enrichment were achieved by fluorescence-activated cell sorting of wild-type bone marrow and spleen.

The Lineage depleted fraction was seeded in a 1:1 ratio with either neutrophil, NK cells, or eosinophils in serum-free stem cell growth medium (20802-0500, SCGM, CellGenix) supplemented with 1% penicillin/streptomycin, 50 ng/ml mouse stem cell factor and 50 ng/ml mouse thrombopoietin. Cultures were kept at 37 °C in a 5% CO_2_ incubator for 16 h until further analysis.

### Enzyme-linked immunosorbent assay

CCL6 levels in cryopreserved bone marrow supernatant, collected from the primary recipients on the day of sacrifice, were measured with a mouse CCL6/C10 DuoSet ELISA kit (DY487, R&D systems) following the manufacturer’s instructions.

### RNA extraction and quality control

HSPCs in the BM, MSCs, and OLCs in the BF were sorted directly into 800 µl TRIzol Reagent (15596018, Thermo Fisher Scientific) and stored at −80 °C until thawed for RNA extraction. Total RNA was isolated following standard protocol. GenElute LPA (56575, Sigma Aldrich) was used as a carrier for ethanol precipitation. After extraction, the RNA pellet was resuspended in 7.5 µl of DEPC-treated water (AM9916, Thermo Fisher Scientific). The quality and quantity of total RNA were examined on a 2100 Bioanalyzer (Agilent) using Agilent RNA 6000 Pico Kit (5067-1513, Agilent).

### RNA sequencing and gene expression profiling

RNA sequencing library preparation was performed as previously described^23,65^. In brief, cDNA was synthesized and amplified using SMART-Seq v4 Ultra Low Input RNA Kit for Illumina Sequencing (634891, Takara Bio) following the manufacturer’s instructions. Amplified cDNA was further processed and the library was created according to TruSeq Nano DNA Library Prep kit (20015964, Illumina) and paired end-sequenced (2 × 75 bp) on the HiSeq 2500 (Illumina). Demultiplexing was performed using the Bcl2Fastq software package (v2.2; Illumina). Adapter sequences and poly-A/T tails were trimmed from the reads using fqtrim (v0.9.7; John Hopkins University, Center for Computational Biology). The preprocessed reads were then aligned using Salmon (v1.3.0)^66^ against the ensemble mouse transcriptome (mm10-build GRCm38.p6) to generate transcript pseudocounts. Differential expression (DE) analysis was performed using DESeq2 (v1.28.1)^67^, followed by multiple testing corrections using the Benjamini–Hochberg procedure to control the false discovery rate (FDR). Genes with corrected *p*-values ≤ 0.05 were considered significant. Genes were ranked based on adaptive shrinkage^68^ of the log2-FoldChange. These ranks were then used in pre-ranked gene-set enrichment analysis (GSEA), which was performed using the Broad-Institute GSEA tool (v4.0.3)^69,70^ with the classic calculation of the Enrichment score and 10,000 permutations based on gene sets. Gene-set libraries C2, C5, and Hallmark (H) for mouse were retrieved with msigdbr (v7.2.1), as extracted from the molecular signature database (v7.2), and selected to have at minimum 15 and at maximum 500 gene members.

### Analysis and comparison of published sequencing data

RNA-sequencing data for young and old mouse HSCs from six public datasets (GSE70657), GSE87631, GSE59114, GSE100426, GSE47817, and GSE109546) were used for comparison. In total four datasets concerned single-cell RNA-seq while the remainder concerned bulk RNA-Seq. Bulk RNA-seq datasets were processed as described in ‘RNA sequencing and gene expression profiling with the exception of pre-ranked GSEA, which was performed with fGSEA (v1.14.0) (Korotkevich et al., bioRxiv 2019) with 1,000,000 permutations, the classic scoring-scheme, and the C2, C5, and H gene-set libraries. DE analysis of single-cell RNA-sequencing data was performed with Seurat (v4.0.0) (Hao et al., bioRxiv 2020) instead of DESeq2. Genes were ranked based on the log10-transformed nominal *p*-value multiplied by the inverted sign of the log2-FoldChange and used as input for pre-ranked GSEA through fGSEA. Differentially expressed genes were filtered on the FDR-adjusted *p*-value (padj) ≤ 0.05 and the pathways analyzed by fGSEA were filtered on padj ≤ 0.25. Genes or pathways present in the overlap between the in-house and one or more public datasets were considered of interest for further investigation.

### Single-cell RNA sequencing

For single-cell RNA sequencing BM and BF were processed as described above. BM was next divided into two parts. The first part was directly stained with differentiated cell markers and subsequently, B and T Cells (B220^+^, CD3^+^), NK Cells, and a myeloid rest fraction (B220^−^, CD3^−^, NKp46^−^ and NK1.1^−^) were separately sorted. The second part of the BM was lineage depleted by magnetic-activated cell sorting (MACS) according to the manufacturer’s protocol (130-090-858, Miltenyi Biotec) and subsequently stained with HSPC markers. HSPC subsets (HSC + MPP (LKS CD48^−^), HPC1 (LKS CD48^+^CD150^−^)) were then sorted separately. The bone fraction was lineage- and CD45 depleted by adding biotinylated anti-CD45.1 and .2 antibodies (respectively, A20, 104, Biolegend) to the above-mentioned MACS protocol. Subsequent niche staining enabled the sort of various populations: endothelial cells (CD31^+^), mesenchymal stromal cells (CD31^−^, Sca1^+^, CD51^+^), osteolineage cells (CD31^−^, Sca1^−^, CD51^+^). In order to enrich for rare populations all subsets mentioned above were sorted separately into Dulbecco’s Modified Eagle Medium (DMEM) + 10% FCS, relatively enriched, pooled together, and then encapsulated for barcoding and cDNA synthesis using the Chromium Single-cell 3′ Reagent kit v3 (10x Genomics) (see Supplementary Fig. 8A). 3’ gene expression library was constructed according to the manufacturer’s recommendations. The quality and quantity of libraries were assessed with an Agilent 2100 Bioanalyzer with 2100 Expert version B.02.11.SI811 software and a High Sensitivity DNA kit. Libraries were sequenced on a NovaSeq 6000 platform (Illumina), paired-end mode, at a sequencing depth of around 25,000 reads per cell, followed by computational alignment using CellRanger (version 3.0.2, 10x Genomics).

Seurat (R package, version 4.0.0)^71^ was used for data preprocessing and downstream analysis. For data preprocessing, datasets were first subjected to quality control steps that included removing doublets (a high ratio of RNA counts vs. feature numbers (>5)) and filtering out apoptotic cells determined by the high transcriptional output of mitochondrial genes (>5% of total). The datasets of each mouse were integrated using the integration function in Seurat followed by linear dimensional reduction. That included scaling of gene expression across all cells, principle component analysis (PCA) on the most variable genes (*k*  =  2000), and unsupervised clustering using a shared nearest neighbor (SNN) modularity optimization-based clustering algorithm (resolution 0.3–1).). To mitigate the effects of cell cycle heterogeneity, cell cycle phase scores were calculated based on canonical markers, and were regressed as described in the Seurat protocol. The data was visualized using uniform manifold approximation and projection for dimension reduction (UMAP)^72^. Non-hematopoietic bone marrow stromal cells (BMSCs) were identified by checking the expression of Cxcl12, Col1a1, and Prrx1 while endothelial cells (ECs) were identified by expression of Vecam and Cd36. These two cell populations were excluded from the analysis. Hematopoietic cell clusters were annotated by assessing the expression of canonical markers associated with particular cell types: HPC_*Ly6a*^*low*^: *Kit*^*+*^, *Ly6a*^*low or* −^, *Cd34*^*high and dim*^; HPC_*Ly6a*^high^: *Kit*^*+*^, *Ly6a*^*high*^, *CD48*^*high*^, *Cd34*^*high*^; MPP: *Kit*+, *Ly6a*^high^, *Cd48*^−^, *Slamf1*^−^, *Cd34*^high^; HSC: *Kit*^*+*^, *Ly6a*^*high*^, *Cd48-*, *Slamf1*^*high*^, *Cd34*^*dim*^ (further confirmed by high *Hes1* and *Meis1* expression); T cells: *Cd3e*^*+*^, *Cd4*^*+*^*/Cd8*^*+*^; NKs: *Cd3e*^*−*^, *Cd8a*^*−*^, *Nkg7*^*+*^; B Lineage: *Cd38*, *Ighm;* Neutrophil (precursors): *Itgam, Ly6g*, *Csf3r*, *Ngp*; Monocyte and DCs: *Itgam Lyz2 Fcgr3*, *Irf8*.

Differentially expressed genes were identified using the non-parametric Wilcoxon rank sum test using the Findmarker function embedded in the Seurat package.

### Statistical analysis

Unless otherwise specified, data presented are mean ± SD. Two-tailed unpaired Student’s *t*-test was used to assess the statistical significance between the two groups. One-way analysis of variance (ANOVA) with Bonferroni correction was used for multiple comparisons.

### Reporting summary

Further information on research design is available in the Nature Portfolio Reporting Summary linked to this article.

## Supplementary information

Supplementary Information

Reporting Summary

Description of Additional Supplementary Files

Supplementary Data 1

## Data Availability

The bulk-RNA sequencing data generated in this study have been deposited in the ArrayExpress database under accession code E-MTAB-10446. The single-cell-RNA sequencing data generated in this study have been deposited in the ArrayExpress database under accession code E-MTAB-12428. Datasets reused in this study: GSE70657, GSE87631, GSE59114, GSE100426, GSE47817, GSE109546). All other data are available in the article and its Supplementary files or from the corresponding author upon reasonable request. Source data are provided with this paper.

## Source data

Source Data
