# Supplementary material for: Myeloid cells promote interferon signaling-associated deterioration of the hematopoietic system
Source: Nat Commun. 2022 Dec 10;13:7657. doi: 10.1038/s41467-022-35318-x (PMC9741615; doi:10.1038/s41467-022-35318-x)
Supplement: Supplementary file 1 — Supplementary Information [file 41467_2022_35318_MOESM1_ESM.pdf]

Myeloid cells drive interferon signaling-associated deterioration of the hematopoietic system

Supplementary Figures and Legends

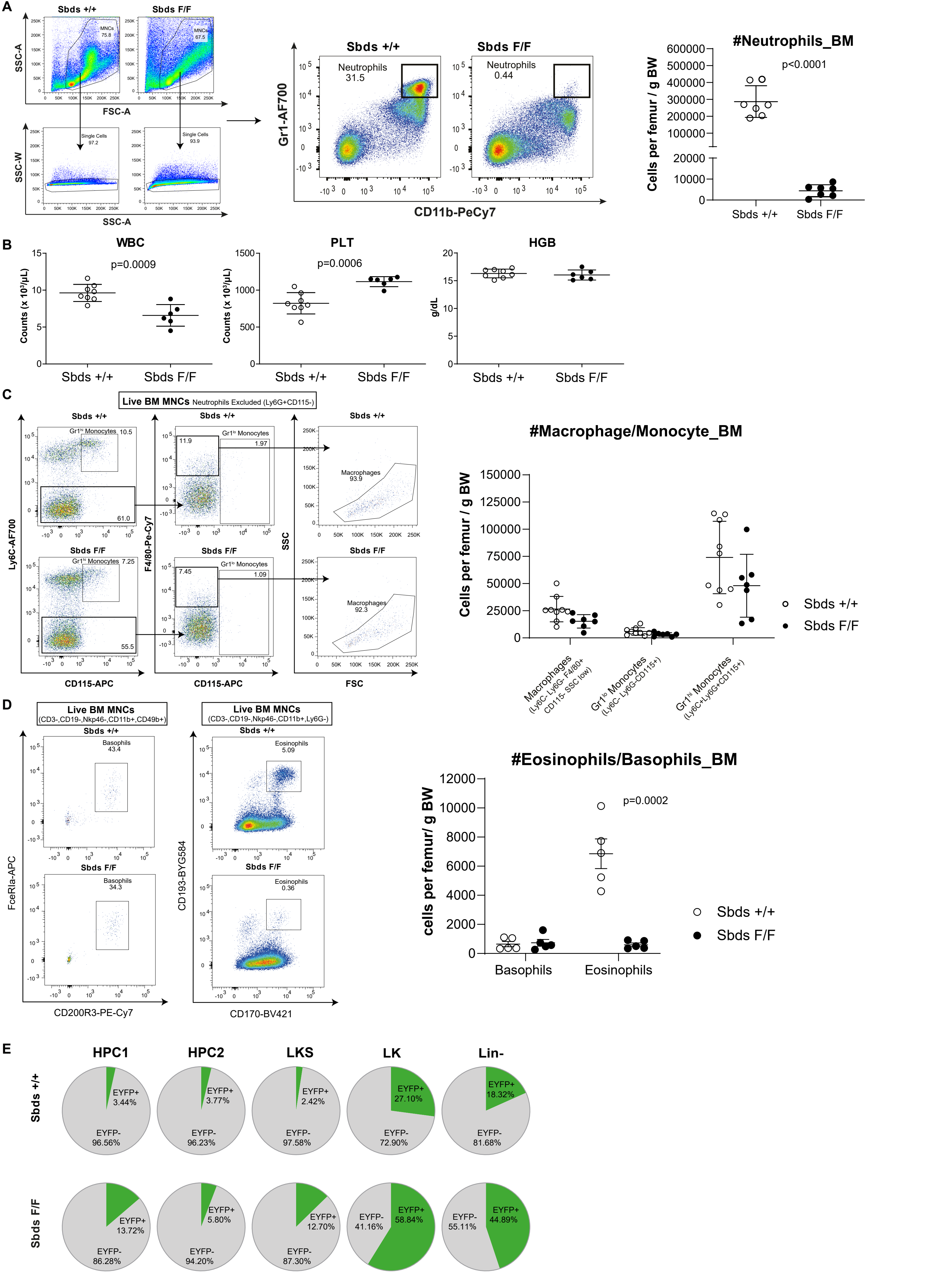

Supplementary Figure 1. A mouse model of profound and sustained neutropenia with genetically intact HSCs.
A. Representative flow cytometry plots of neutrophil (Gr1+/CD11b+) frequencies and absolute neutrophil counts in the bone marrow (n=7). B. Peripheral blood counts and hemoglobin levels ($n=8^{+/+},6^{F/F}$). C. Absolute monocyte and macrophage counts in the bone marrow with representative gating strategy ($n=9^{+/+},7^{F/F}$). D. Representative gating strategy (left panel) and absolute eosinophil and basophil counts in the bone marrow (right panel) (n=5). E. The percentage of EYFP+ cells in hematopoietic progenitor subsets.
Data are mean ± S.D. Two-sided unpaired t-test was performed for statistical analysis.Source data are provided as a Source Data file.

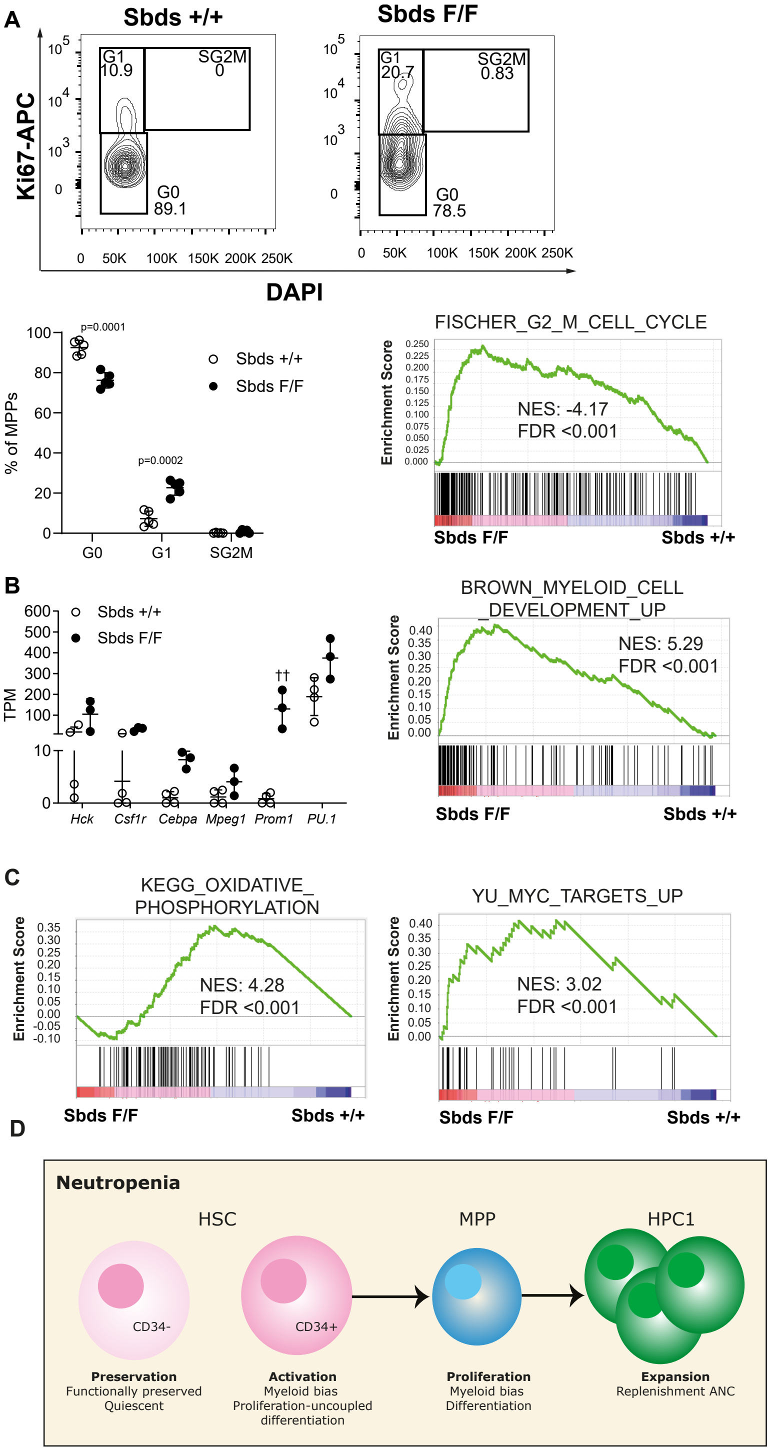

Supplementary Figure 2. MPPs display increased cycling, myeloid priming and metabolic activation in neutropenia.
Data are from MPPs from neutropenic ($Sbds^{F/F}$) mice compared to MPPs from control ($Sbds^{+/+}$) mice at 4 months after primary transplant. A. Increased cycling of MPPs in neutropenia as demonstrated by Ki67/DAPI staining (n=5) and enrichment of cell cycle associated transcriptional programs. B. Increased expression of myeloid-differentiation related transcripts and enrichment of myeloid differentiation transcriptional programs (GSEA) in MPPs from neutropenic mice ($n=4^{+/+},3^{F/F}$). C. Enrichment of transcriptional programs consistent with increased oxidative phosphorylation and Myc activation. D. Schematic representation of HSPC response to neutropenia.
Data are mean ± S.D. Two-sided unpaired t-test was performed for statistical analysis. TPM: Transcripts Per Kilobase Million. ††† FDR < 0.001, †† FDR < 0.01, † FDR < 0.05. NES, Normalized p value, and FDR value of each gene set are as listed. GSEA: gene sets enrichment analysis. NES: normalized enrichment score. FDR: false discovery rate. Source data are provided as a Source Data file.

F/F

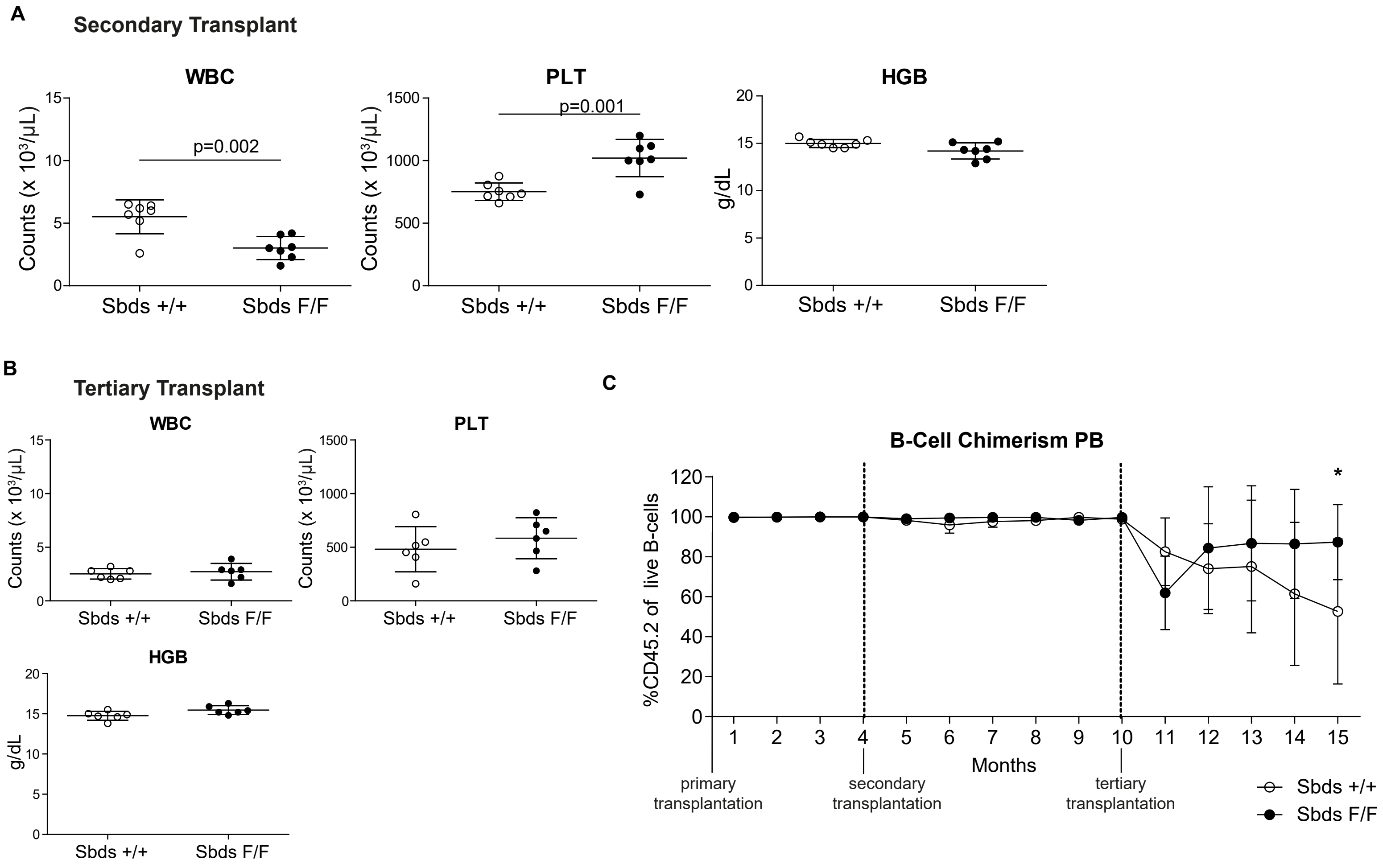

Supplementary Figure 3. Peripheral blood counts and chimerism after serial transplantation.
A. Peripheral blood counts and hemoglobin levels of secondary recipients in serial transplantation (n=7). B. Peripheral blood counts and hemoglobin levels of tertiary recipients in serial transplantation (n=6). C. Enhanced long-term, serial lymphoid repopulation ability of HSCs exposed to sustained neutropenia (related to figure 3G as shown in percentage of CD45.2+ B-cells in peripheral blood (n=5-6).
Data are mean ± S.D.. A-B: Two-sided unpaired t-test was performed for statistical analysis. C: One-way analysis of variance (ANOVA) with Bonferroni correction was used for multiple comparisons; *** $p < 0.001$, ** $p < 0.01$, * $p < 0.05$. Source data are provided as a Source Data file

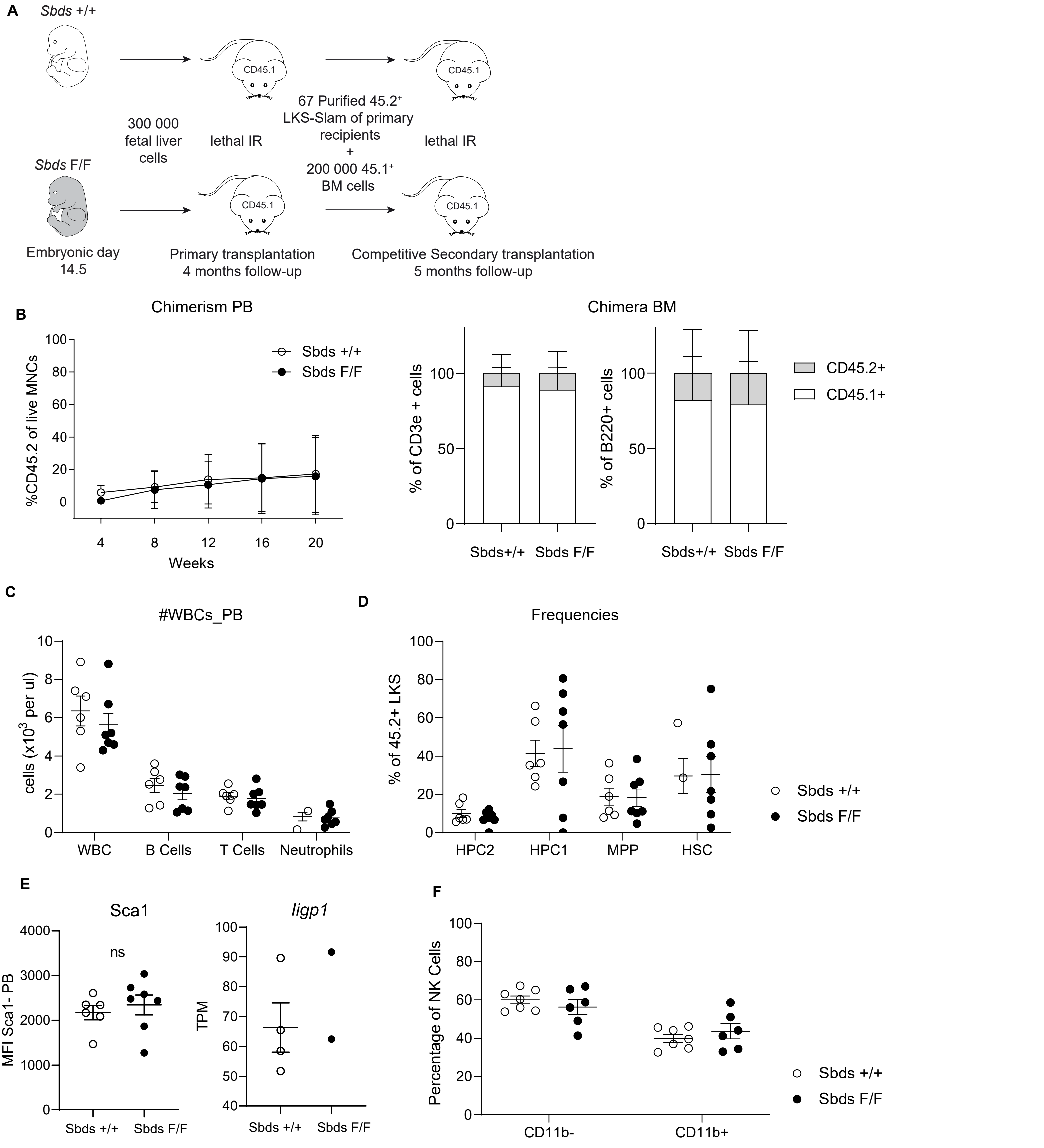

Supplementary Figure 4. Correction of neutropenia in a competitive transplant setting reverses the hematopoietic phenotype of Cebpacre/+ SbdsF/F mice.
Secondary competitive transplant with purified HSCs (LKS-Slam). A. Schematic representation of competitive transplant. B. CD45.2 chimerism in peripheral blood and lymphoid (CD3e+ T-cell and B220+ B-cell) chimerism in the bone marrow. C. Normalization of neutrophil counts in the competitive setting. D. Normalization of LKS subset frequencies. B-D: (n=$6^{+/+}$,$7^{F/F}$). E. Normalization of Sca1 (n=$6^{+/+}$,$7^{F/F}$) and Iigp1 (n=$4^{+/+}$,$2^{F/F}$) in CD45.2+ HSC-Slam after correction of neutropenia in competitive transplant. F. Normalization of NK cell-activation skewing in competitive setting (n=$6^{+/+}$,$7^{F/F}$).
Data are mean ± SEM. Two-sided unpaired t-test was performed for statistical analysis. TPM: Transcripts Per Kilobase Million. Source data are provided as a Source Data file

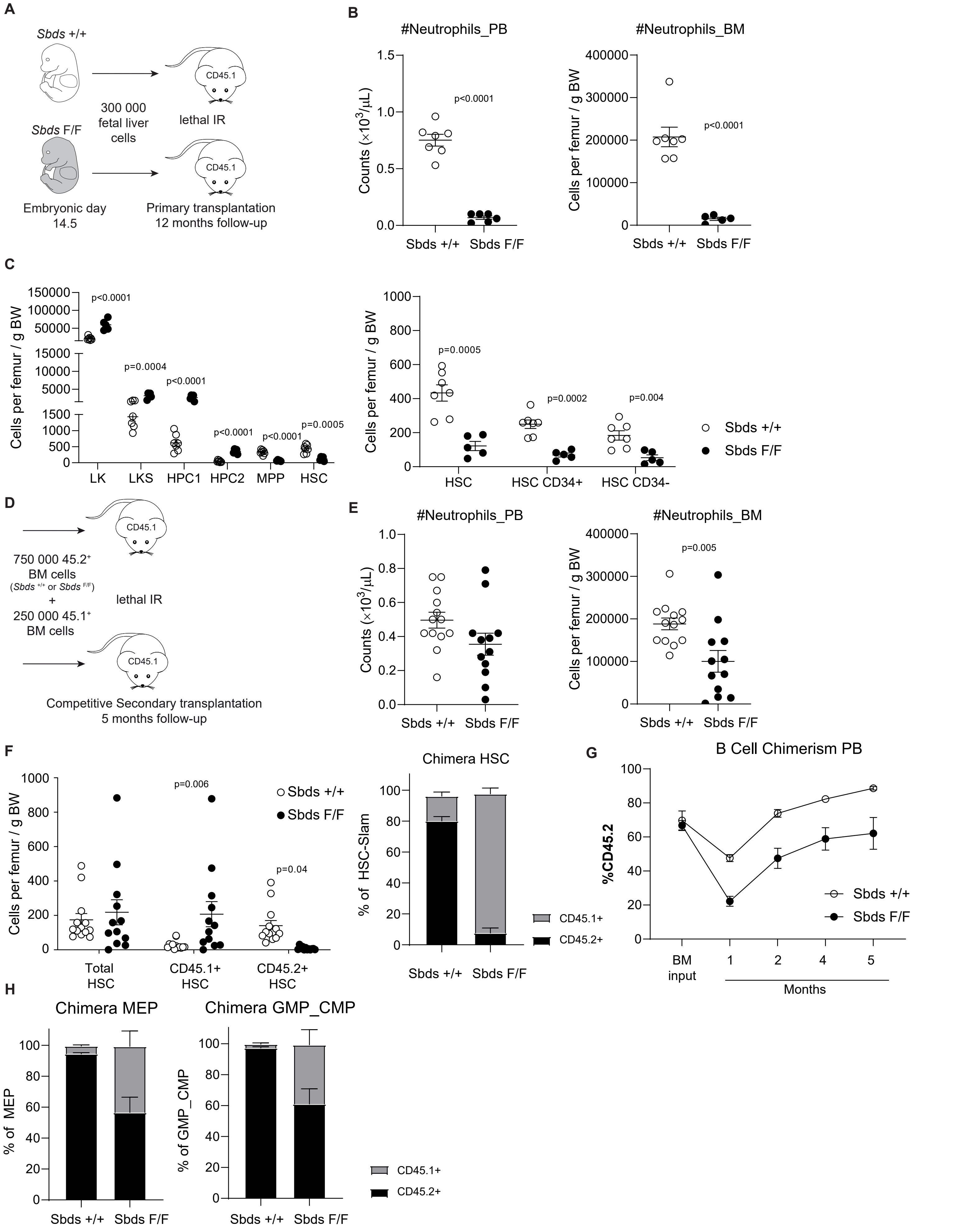

Supplementary Figure 5. HSC numbers and function upon long-term follow-up of neutropenic mice.
A. Schematic representation of the experimental design of transplantation with long-term follow-up. Neutropenic mice and controls were sacrificed 12 months after transplantation of Cebpacre/+ Sbds$^{F/F}$ or +/+ cells. B. Absolute number of peripheral blood (n=7$^{+/+}$,6$^{F/F}$) and bone marrow (n=7$^{+/+}$,5$^{F/F}$) neutrophils. C. Increased numbers of progenitors and reduced numbers of HSCs in neutropenic mice (n=7$^{+/+}$,5$^{F/F}$). D. Schematic representation of competitive transplant. Bone marrow cells from neutropenic or control mice (CD45.2) were transplanted competitively with CD45.1 bone marrow cells in a 3:1 ratio. E. Reduced neutrophil numbers in mice transplanted with bone marrow cells from neutropenic mice (n=13$^{+/+}$,12$^{F/F}$). F. Absolute number of immunophenotypic (LKS-Slam) HSCs (left panel) and frequency of CD45.2 cells within HSC compartment (right panel) (n=13$^{+/+}$,12$^{F/F}$). G. CD45.2 chimerism in peripheral blood. Note that CD45.2 HSCs from neutropenic mice constitute < 10% of immunophenotypic HSCs (Figure E, right panel), yet contribute to approximately 60% of multileage hematopoietic output as indicated by the B-cell chimerism (G) and contribution to MEP and CMP progenitor pools (H) (n=13$^{+/+}$,12$^{F/F}$).
Data are mean ± SEM. Two-sided unpaired t-test was performed for statistical analysis. Source data are provided as a Source Data file.

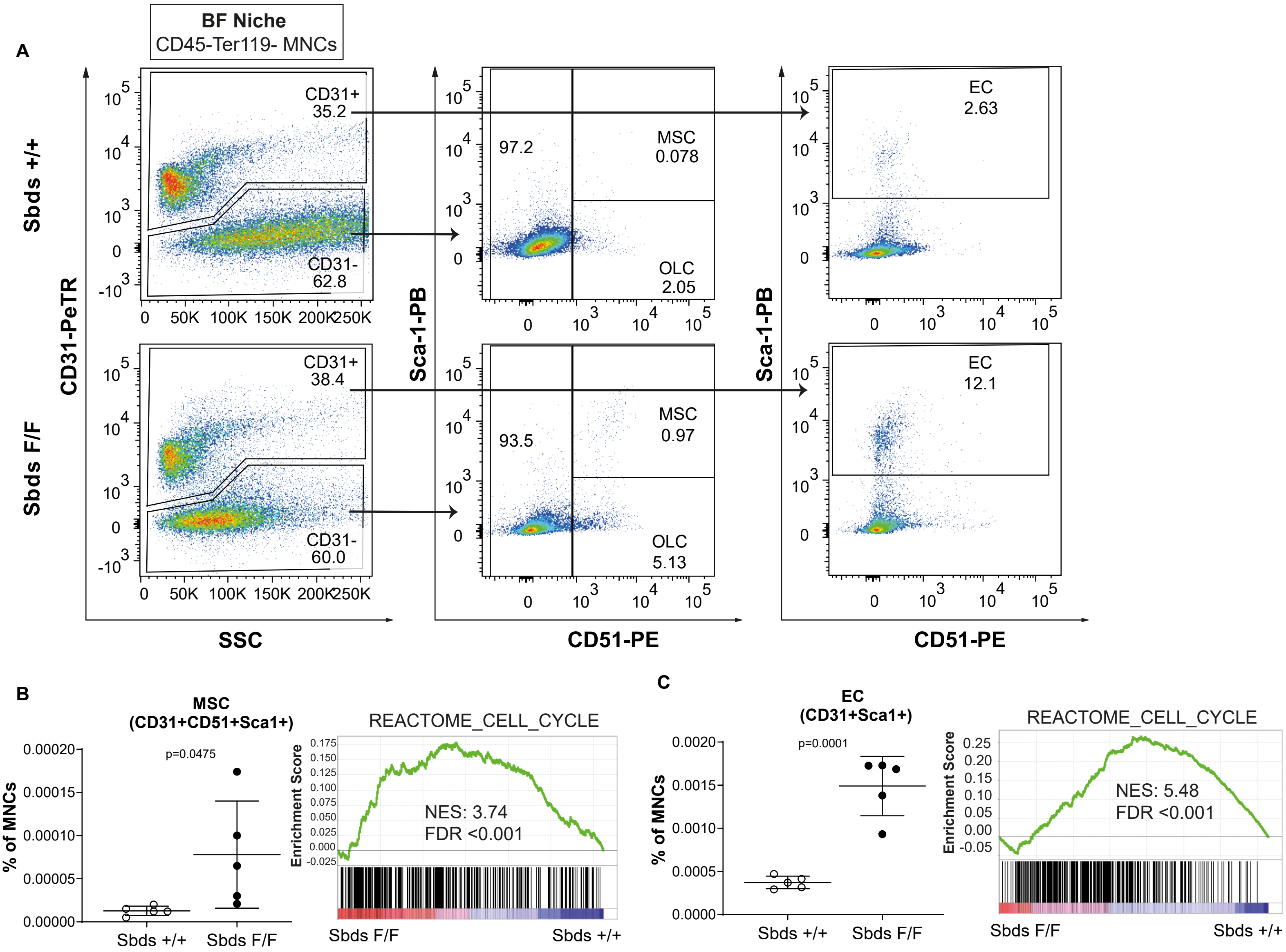

Supplementary Figure 6. Remodeling of HSPC niches in neutropenia.
A. Representative flow cytometry gating of niche populations in collagenased bone fractions of neutropenic vs. control mice after the primary transplant is shown. B. Relative expansion and transcriptional wiring consistent with proliferation in mesenchymal niche cells (n=5). C. Relative expansion and transcriptional wiring consistent with proliferation in endothelial niche cells (n=5).
Data are mean ± S.D. Two-sided unpaired t-test was performed for statistical analysis. OLC: osteolineage cell, EC: endothelial cell, MSC: mesenchymal cell. NES and FDR value of each gene set are as listed. GSEA: gene sets enrichment analysis. NES: normalized enrichment score. FDR: false discovery rate. Source data are provided as a Source Data file.

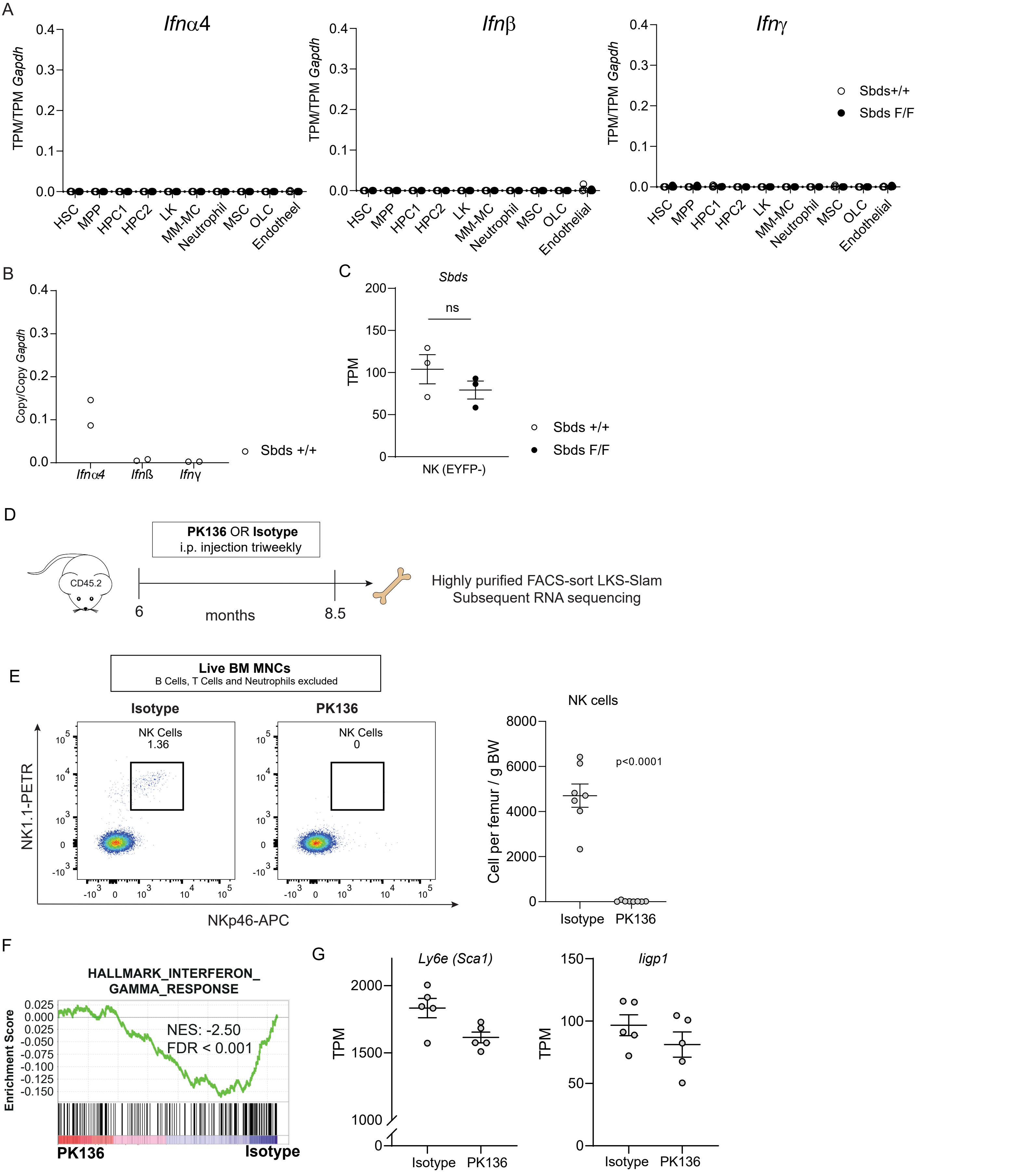

Supplementary Figure 7. NK cells, but not neutrophils, induce IFN signaling in HSCs.
A. Lack of expression of *interferon alpha4* (*Ifnα4*), *interferon beta* (*Ifnβ*) and *interferon gamma* (*Ifnγ*) throughout the myeloid lineage, from HSC to mature neutrophils isolated from the $Sbds^{F/F}$ and $Sbds^{+/+}$ bone marrow (n=3-4). B. Ifnα4, Ifnβ and Ifnγ expression in BM eosinophils (n=2). C. Sbds transcript levels within bone marrow EYFP- NK cells. D-H. In vivo depletion of NK cells mitigates transcriptional IFN signaling in HSCs (n=3). D. Scheme of experimental set up. E. Depletion of NK cells in the bone marrow at 10 weeks, representative flow cytometry plots of NK cell frequencies and absolute NK cell counts in BM are shown (n= $7^{+/+}$,$8^{F/F}$). F. Downregulation of transcriptional type II IFN signaling upon NK cell depletion in HSC-Slam. G. Expression of *Sca1* and *Iigp1* in HSC-Slam after NK cell depletion (n=5).
Data are mean ± SEM. Two-sided unpaired t-test was performed for statistical analysis. TPM: Transcripts Per Kilobase Million. MM-MC: metamyelocyt-myelocyt. Source data are provided as a Source Data file.

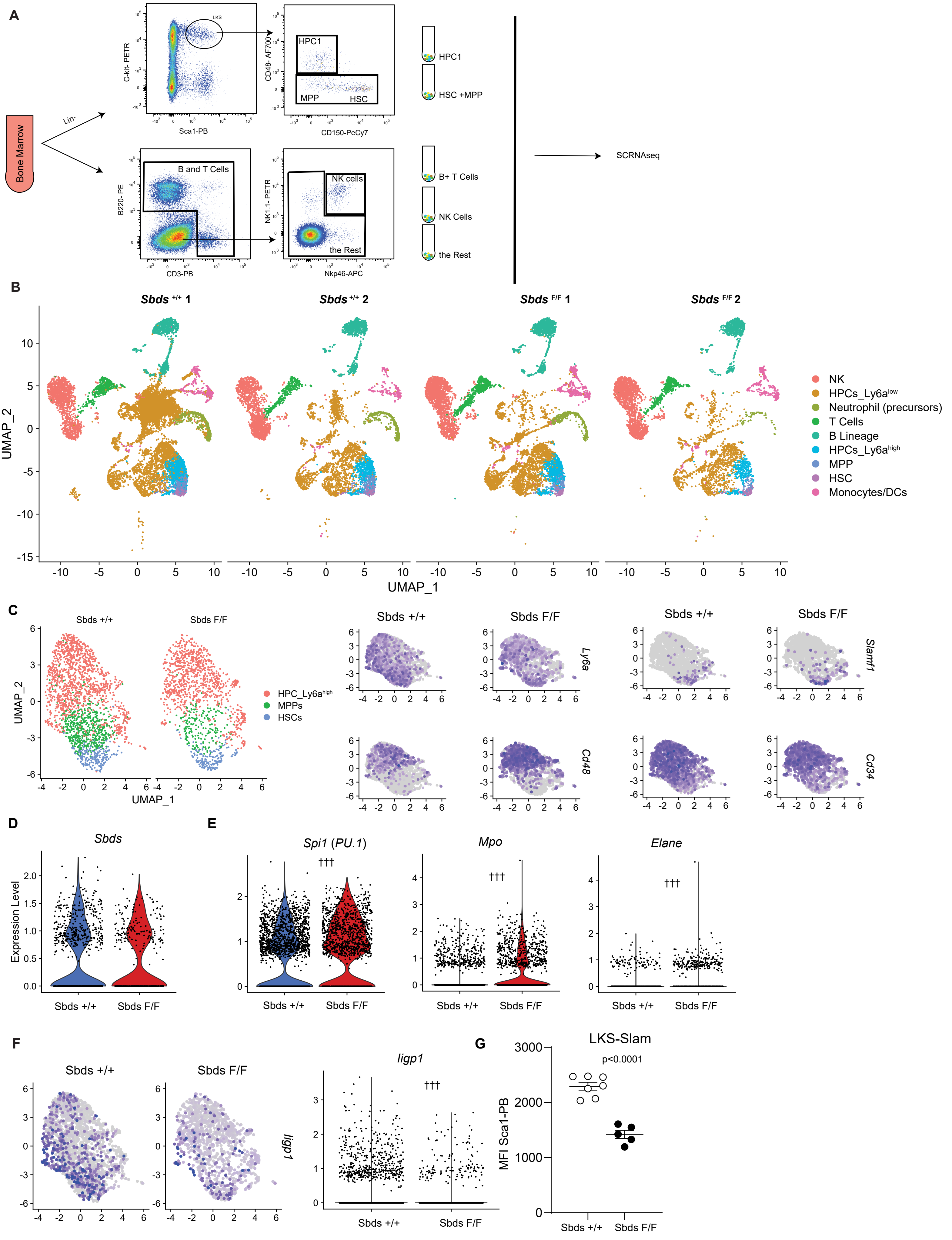

Supplementary Figure 8. HSPC characteristics upon long-term follow-up of neutropenic mice.
Neutropenic mice and controls were sacrificed 12 months after transplantation of *Cebpacre*/+ *Sbds*$^{F/F}$ or $^{+/+}$ cells. A. FACS gating strategy for sorting of hematopoietic cell populations from bone marrow and bone fraction for scRNA sequencing. Bone marrow cells were subsorted into HSC+MPP (LKS CD48$^-$), HPC1 (LKS CD48$^+$CD150$^-$), B and T Cells (B220$^+$, CD3$^+$), NK Cells and a myeloid rest fraction (B220$^-$, CD3$^-$, NKp46andNK1.1$^-$). The subsorted fraction were pulled together to obtain robust representation of all bone marrow cell types in the scRNAseq data. B. Uniform Manifold Approximation and Projection (UMAP) plot of bone marrow mononuclear cells, representing 13124 cells from two neutropenic mice and 18148 cells from two control mice. C. UMAP plots of HSPCs discerning HSC, MPP and HPC_Ly6a^{high} subsets based on marker expression (right panels). HSCs are identified within the HSPC population by higher *Slamf1* expression and no detectable expression of *Cd34* and *Cd48*. D. *Sbds* expression indicating Sbds proficiency in HSPCs from neutropenic mice. E. Transcriptional myeloid priming of HSPCs in neutropenia. Expression of various myeloid commitment genes in HSPCs is plotted. F. Reduced expression of the canonical IFN transcriptional target, *Iigp1* in HSPCs from neutropenic mice (B-F: n=2). G. Reduced Sca1 protein expression (as assessed by flow-cytometry) in HSCs from neutropenic mice (n=7$^{+/+}$,5$^{F/F}$).
Data are mean ± SEM. Two-sided unpaired t-test was performed for statistical analysis. ††† FDR < 0.001, †† FDR < 0.01, † FDR < 0.05. FDR: false discovery rate. Source data are provided as a Source Data file.

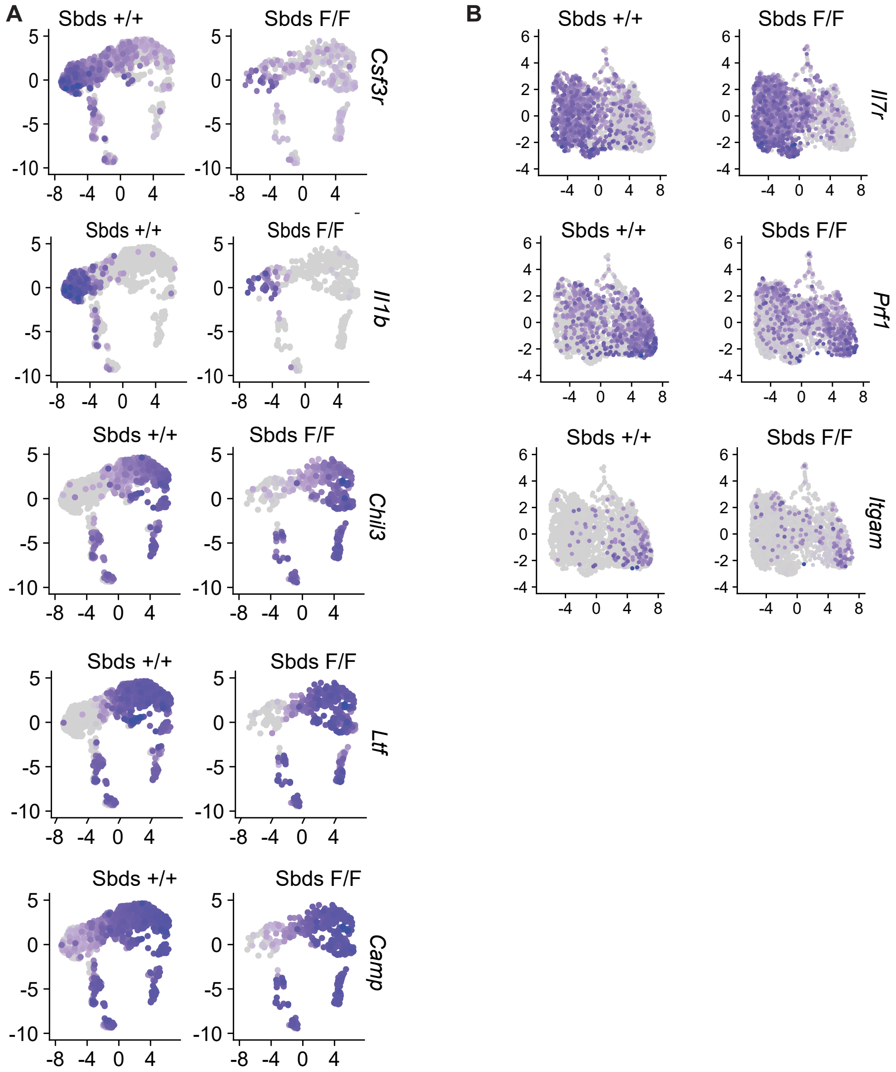

Supplementary Figure 9. Delineation of NK and myeloid cell heterogeneity in neutropenic mice.
A. Depicted genes used to subset the neutrophil (precursor) populations. Transcripts associated with an immature fraction: *Chi3, Ltf and Camp* and mature fraction: *Il1b and Csf3r.* B. Transcripts used to subcluster NK population into an immature a nd a mature fraction based on expression of *Il7r, Prf1* and *Itgam* (n=2).

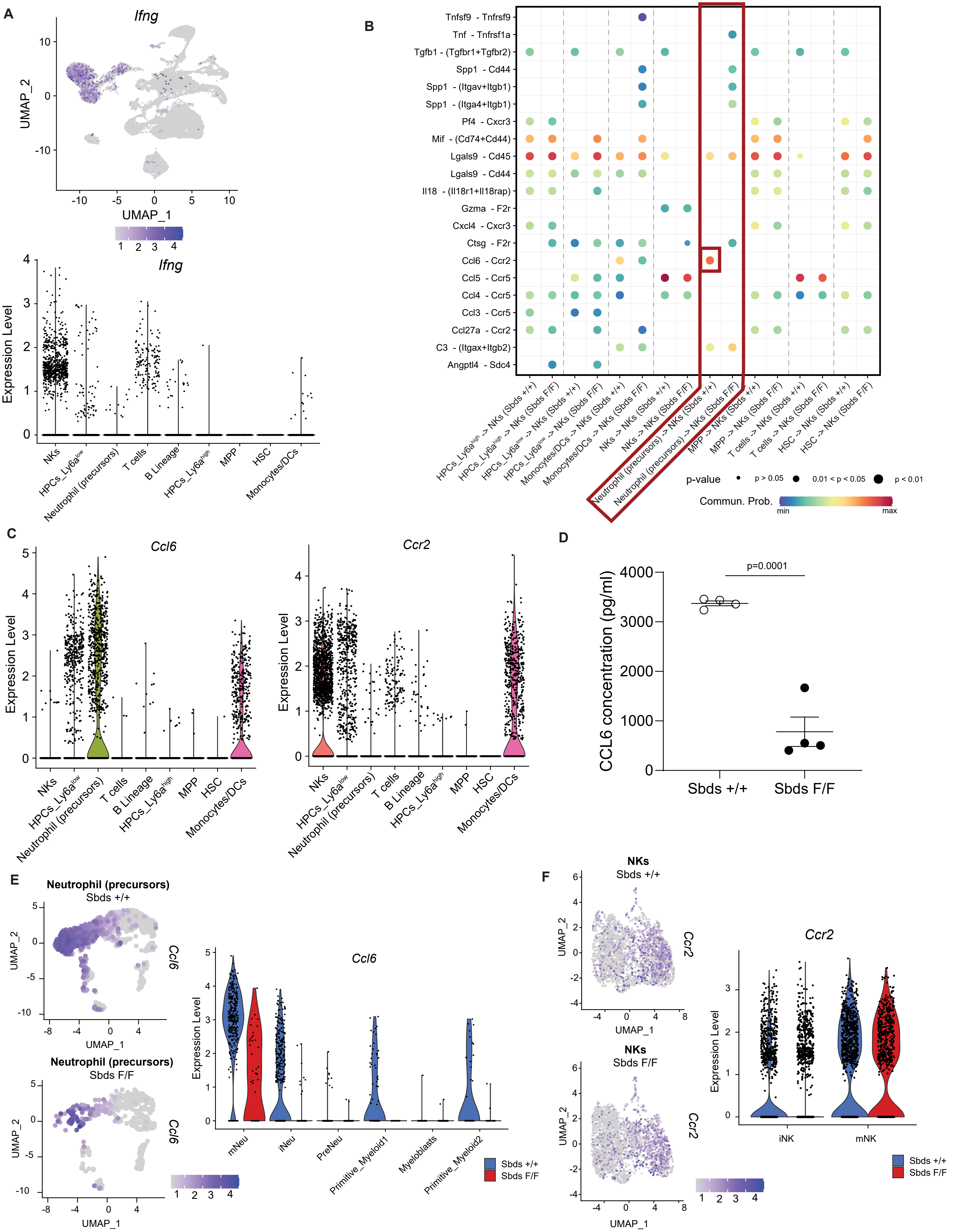

Supplementary Figure 10. Delineation of intercellular signaling between bone marrow cells in neutropenic mice.
A. UMAP and violin plots identifying NK cells as cells with the highest *interferon gamma (Ifng)* expression within the murine bone marrow. B. Predicted ligand-receptor interactions between NK cells and other cell types. Color scale and dot size represent probability and p-value of interactions, respectively. Note the absence of CCL6-CCR2 signaling between myeloid cells and NK cells in neutropenic mice. C. Confirmation in $Sbds^{+/+}$ mice that *Ccl6* is predominately expressed by the myeloid fraction, and *Ccr2* is predominately expressed by NK cells (A-C: n=2). D. CCL6 protein levels are significantly higher in bone marrow supernatant of $Sbds^{+/+}$ mice than $Sbds^{F/F}$ (n=4). Data are mean ± SEM. Two-sided unpaired t-test was performed for statistical analysis. E. *Ccr2* is specifically expressed in the mature subset of NK cells. F. *Ccl6* is specifically expressed immature neutrophils with significant lower expression in neutropenic mice (E-F: n=2). mNeu: mature BM neutrophil. iNeu: immature BM neutrophil. Pre-Neu: precursor neutrophil. iNK: immature NK celll. mNK: mature NK cell. DC: dendritic cell.
Source data are provided as a Source Data file.
