# Supplementary material for: Myeloid cells promote interferon signaling-associated deterioration of the hematopoietic system
Source: Nat Commun. 2022 Dec 10;13:7657. doi: 10.1038/s41467-022-35318-x (PMC9741615; doi:10.1038/s41467-022-35318-x)
Supplement: Supplementary file 2 — Reporting Summary [file 41467_2022_35318_MOESM2_ESM.pdf]

nature portfolio

Corresponding author(s): Prof. dr. M.H.G.P. Raaijmakers

Last updated by author(s): Nov 13, 2022

Reporting Summary

Nature Portfolio wishes to improve the reproducibility of the work that we publish. This form provides structure for consistency and transparency in reporting. For further information on Nature Portfolio policies, see our Editorial Policies and the Editorial Policy Checklist.

Statistics

For all statistical analyses, confirm that the following items are present in the figure legend, table legend, main text, or Methods section.

n/a	Confirmed	
☐	☒	The exact sample size (*n*) for each experimental group/condition, given as a discrete number and unit of measurement
☐	☒	A statement on whether measurements were taken from distinct samples or whether the same sample was measured repeatedly
☐	☒	The statistical test(s) used AND whether they are one- or two-sided<br>*Only common tests should be described solely by name; describe more complex techniques in the Methods section.*
☒	☐	A description of all covariates tested
☐	☒	A description of any assumptions or corrections, such as tests of normality and adjustment for multiple comparisons
☐	☒	A full description of the statistical parameters including central tendency (e.g. means) or other basic estimates (e.g. regression coefficient) AND variation (e.g. standard deviation) or associated estimates of uncertainty (e.g. confidence intervals)
☐	☒	For null hypothesis testing, the test statistic (e.g. *F*, *t*, *r*) with confidence intervals, effect sizes, degrees of freedom and *P* value noted<br>*Give P values as exact values whenever suitable.*
☒	☐	For Bayesian analysis, information on the choice of priors and Markov chain Monte Carlo settings
☒	☐	For hierarchical and complex designs, identification of the appropriate level for tests and full reporting of outcomes
☒	☐	Estimates of effect sizes (e.g. Cohen's *d*, Pearson's *r*), indicating how they were calculated

Our web collection on statistics for biologists contains articles on many of the points above.

Software and code

Policy information about availability of computer code

Data collection: No software was used for collection of data.

Data analysis: Data analysis and plotting were done using GraphPad Prism 8.

Bulk RNA seq:
The preprocessed reads were aligned using Salmon (v1.3.0) against the ensemble mouse transcriptome (mm10-build GRCm38.p6) to generate transcript pseudocounts. Differential Expression (DE) analysis was performed using DESeq2 (v1.28.1), followed by multiple testing correction using the Benjamini-Hochberg procedure to control the False Discovery Rate (FDR). Genes with corrected p-values 0.05 were considered significant. Genes were ranked based on adaptive shrinkage of the log2-FoldChange. These ranks were then used in pre-ranked gene-set enrichment analysis (GSEA), which was performed using the Broad-Institute GSEA tool (v4.0.3) with classic calculation of the Enrichment Score and 10.000 permutations based on gene-sets. Gene-set libraries C2, C5 and Hallmark (H) for mouse were retrieved with msigdbr (v7.2.1), as extracted from the molecular signature database (v7.2), and selected to have at minimum 15 and at maximum 500 gene members.

Single Cell RNA-seq:
Seurat (R package, version 4.0.0) was used for data preprocessing and downstream analysis. For data preprocessing, datasets were first subjected to quality control steps that included removing doublets (a high ratio of RNA counts vs. feature numbers (> 5)) and filtering out apoptotic cells determined by high transcriptional output of mitochondrial genes (>5% of total). The datasets of each mouse were integrated using the integration function in Seurat followed by linear dimensional reduction. That included scaling of gene expression across all cells, principle component analysis (PCA) on the most variable genes (k=2,000) and unsupervised clustering using a shared nearest neighbor (SNN) modularity optimization-based clustering algorithm (resolution 0.3–1).). To mitigate the effects of cell cycle heterogeneity, cell cycle phase scores were calculated based on canonical markers, and were regressed as described in Seurat protocol. The data was visualized using Uniform Manifold Approximation and Projection for Dimension Reduction (UMAP). Non-hematopoietic bone marrow stromal cells (BMSCs) were identified by checking expression of Cxcl12, Col1a1 and Prrx1 while endothelial cells (ECs) were identified by expression of Vecam and

Cd36. These two cell populations were excluded from the analysis. Hematopoietic cell clusters were annotated by assessing expression of canonical markers associated with particular cell types: HPC_Ly6alow: Kit+, Ly6alow or -, Cd34high and dim; HPC_Ly6ahigh: Kit+, Ly6ahigh, CD48high, Cd34high; MPP: Kit+, Ly6ahigh, Cd48-, Slamf1-, Cd34high; HSC: Kit+, Ly6ahigh, Cd48- , Slamf1high, Cd34dim (further confirmed by high Hes1 and Meis1 expression); T cells: Cd3e+, Cd4+/Cd8+; NKs: Cd3e-, Cd8a-,Nkg7+; B Lineage: Cd38, Ighm; Neutrophil (precursors): Itgam, Ly6g , Csf3r , Ngp; Monocyte and DCs: Itgam Lyz2 Fcgr3, Irf8.
Differential expressed genes were identified using the non-parametric Wilcoxon rank sum test using the Findmarker function embedded in the Seurat package.

For manuscripts utilizing custom algorithms or software that are central to the research but not yet described in published literature, software must be made available to editors and reviewers. We strongly encourage code deposition in a community repository (e.g. GitHub). See the Nature Portfolio guidelines for submitting code & software for further information.

Data

Policy information about availability of data

All manuscripts must include a data availability statement. This statement should provide the following information, where applicable:

- Accession codes, unique identifiers, or web links for publicly available datasets
- A description of any restrictions on data availability
- For clinical datasets or third party data, please ensure that the statement adheres to our policy

Primary Datasets:
- The bulk-RNA sequencing data generated in this study have been deposited in the ArrayExpress database under accession code E-MTAB-10446 [https://www.ebi.ac.uk/biostudies/arrayexpress/studies/E-MTAB-10446].
- The single-cell-RNA sequencing data generated in this study have been deposited in the ArrayExpress database under accession code E-MTAB-12428 [https://www.ebi.ac.uk/biostudies/arrayexpress/studies/E-MTAB-12428].

Referenced Datasets:
-GSE70657 [https://www.ncbi.nlm.nih.gov/geo/query/acc.cgi?acc=GSE70657]
-GSE87631 [https://www.ncbi.nlm.nih.gov/geo/query/acc.cgi?acc=GSE87631]
-GSE59114 [https://www.ncbi.nlm.nih.gov/geo/query/acc.cgi?acc=GSE59114]
-GSE100426 [https://www.ncbi.nlm.nih.gov/geo/query/acc.cgi?acc=GSE100426]
-GSE47817 [https://www.ncbi.nlm.nih.gov/geo/query/acc.cgi?acc=GSE47817]
-GSE109546 [https://www.ncbi.nlm.nih.gov/geo/query/acc.cgi?acc=GSE109546])

- All RNA seq downstream analyses are derived from these raw data
- No restriction.

Field-specific reporting

Please select the one below that is the best fit for your research. If you are not sure, read the appropriate sections before making your selection.

☒ Life sciences ☐ Behavioural & social sciences ☐ Ecological, evolutionary & environmental sciences

For a reference copy of the document with all sections, see nature.com/documents/nr-reporting-summary-flat.pdf

Life sciences study design

All studies must disclose on these points even when the disclosure is negative.

Sample size	In vivo experiments:<br>We did not perform a calculation on the sample size. The group size was based on our own and published experiences that meaningful differences in key HSC and hematopoietic parameters can be assessed in such a cohort. (Beerman et al., PNAS, 2010; Essers et al., Nature, 2009; Kenswill et al., Cell Stem Cell, 2021)<br><br>Ex vivo experiments:<br>Sample size was not predetermined. At least three independent experimental replicates were used to provide statistical significance for possible observed differences.
Data exclusions	No data were excluded.
Replication	All findings have been reproduced in independent experiments and multiple animals as indicated in the manuscript figures.
Randomization	Mice were allocated BM-genotype and treatment groups randomly.
Blinding	Seeing as all experiments were performed by researchers and care takers, it was not possible to blind these studies.

Reporting for specific materials, systems and methods

We require information from authors about some types of materials, experimental systems and methods used in many studies. Here, indicate whether each material, system or method listed is relevant to your study. If you are not sure if a list item applies to your research, read the appropriate section before selecting a response.

Materials & experimental systems

n/a	Involved in the study	
☐	☒	Antibodies
☒	☐	Eukaryotic cell lines
☒	☐	Palaeontology and archaeology
☐	☒	Animals and other organisms
☒	☐	Human research participants
☒	☐	Clinical data
☒	☐	Dual use research of concern

Methods

n/a	Involved in the study	
☒	☐	ChIP-seq
☐	☒	Flow cytometry
☒	☐	MRI-based neuroimaging

Antibodies

Antibodies used

Ly-6G/Ly-6C (Gr-1) AF700 RB6-8C5 Biolegend Cat #108422
Ly-6G/Ly-6C (Gr-1) APC RB6-8CS Biolegend Cat #108412
Ly-6G/Ly-6C (Gr-1) Biotin RB6-8C5 BD Bioscience Cat #13-5931-82
CD11b PE-Cy7 M1/70 Biolegend Cat #101216
CD11b APC-CY7 M1/70 Biolegend Cat #101226
Ly6G AF700 1A8 Biolegend Cat #127622
Ly6C BV510 HK1.4 Biolegend Cat #128033
CD115 APC AFS98 Biolegend Cat #135510
B220 eFluor450 RA3-6B2 eBioscience Cat #48-0452-82
B220 PE RA3-6B2 BioLegend Cat #103208
F4/80 PE-Cy7 BM8 SONY Biotechnology Cat #1215570
CD3e BV510 145-2C11 BD Bioscience Cat #563024
CD3 eFlour450 17A2 eBioscience Cat #48-0032-82
CD43 PE-Cy7 Ly-48 Biolegend Cat #121218
CD27 BV510 LG.3A10 Biolegend Cat #124229
NK-1.1 PE/Dazzle™ 594 PK136 Biolegend Cat #108748
NKp46 eFlour660 29A1.4 eBioscience Cat #50-3351-82
CD11b Biotin M1/70 BD Biosciences Cat #553309
Ter119 Biotin TER-119 BD Biosciences Cat #553672
CD3e Biotin 145-2C11 BD Biosciences Cat #553060
CD4 Biotin GK1.5 BD Biosciences Cat #553045
CD8 Biotin 53-6.7 BD Biosciences Cat #553029
B220 Biotin RA3-6B2 BD Biosciences Cat #553085
Streptavidin Pacific Orange Life Technologies Cat #S32365
Sca1 Pacific Blue E13-161.7 Biolegend Cat #122520
CD48 AF700 HM48-1 Biolegend Cat #103426
CD150 PE-Cy7 TC15-12F12.2 Biolegend Cat #115914
c-Kit APC 2B8 BD Biosciences Cat #553356
c-Kit PE-CF594 2B8 BD Biosciences Cat #562417
CD16/32 APC-Cy7 2.4G2 BD Biosciences Cat #560541
CD34 eFluor450 RAM34 eBioscience Cat #48-0341-82
CD34 eFluor660 RAM34 eBioscience Cat #50-0341-82
CD45.1 APC-Cy7 A20 Biolegend Cat #110716
CD45.2 APC-Cy7 104 Biolegend Cat #109824
Ter119 BV510 TER-119 Biolegend Cat #116237
CD51 PE RMV-7 Biolegend Cat #104106
CD31 PE-CF594 MEC 13.3 BD Biosciences Cat #563616
CD140α APC APA5 eBioscience Cat #17-1401-81
CD45.1 PE A20 Biolegend Cat #110708
CD45.2 APC 104 Biolegend Cat #109814
CD45.2 APC-Cy7 104 Biolegend Cat #109824
CD45.1 Biotin A20 Biolegend Cat #110704
CD45.2 Biotin 104 Biolegend Cat #109804
NK-1.1 Biotin PK136 Biolegend Cat #108704
CD19 Biotin 6D5 Biolegend Cat #115503
CD11b BB700 (PerCP Cy5.5) M1/70 Biolegend Cat #101228

CD193 PE J073E5 Biolegend Cat #144505
CD170 (Anti-Siglec-F) BV421 S17007L Biolegend Cat #155509
CD49b PE HMα2 Biolegend Cat #103506
CD200R3 PE-Cy7 Ba13 Biolegend Cat #142211
Anti-FcεRIα APC MAR-1 Biolegend Cat #134315
7-AAD BB700 (PerCP Cy5.5) Biolegend Cat #420404
Zombie NIR™ Fixable Viability Kit APC-Cy7 Biolegend Cat #423105
LIVE/DEAD™ Fixable Aqua Dead Cell Stain Kit Invitrogen Cat #L34957

Validation

Ly-6G/Ly-6C (Gr-1) AF700 Validation: https://www.nature.com/articles/ni1244 DOI: 10.1038/ni1244 RRID:AB_2137487
Ly-6G/Ly-6C (Gr-1) APC Validation: https://www.ncbi.nlm.nih.gov/pmc/articles/PMC1794052/ DOI: 10.1182/blood-2006-03-013771 RRID:AB_313377
Ly-6G/Ly-6C (Gr-1) Biotin Validation: https://www.ncbi.nlm.nih.gov/pmc/articles/PMC6286615/ DOI: 10.1186/s40364-018-0149-4 RRID:AB_466800
CD11b PE-Cy7 Validation: https://www.ncbi.nlm.nih.gov/pmc/articles/PMC2515136/ DOI: 10.1182/blood-2008-01-134304 RRID:AB_312799
CD11b APC-CY7 Validation: https://www.jimmunol.org/content/180/1/609.long DOI: 10.4049/jimmunol.180.1.609 RRID:AB_830642
Ly6G AF700 Validation: https://www.ncbi.nlm.nih.gov/pmc/articles/PMC3169908/ DOI: 10.4049/jimmunol.1000383 RRID:AB_10643269
Ly6C BV510 Validation: https://www.ncbi.nlm.nih.gov/pmc/articles/PMC7415677/ DOI: 10.1016/j.cell.2020.06.021 RRID:AB_2562351
CD115 APC Validation: https://www.ncbi.nlm.nih.gov/pmc/articles/PMC5317374/ DOI: 10.1038/nature20131 RRID:AB_2085221
B220 eFluor450 Validation: https://www.ncbi.nlm.nih.gov/pmc/articles/PMC7756897/ DOI: 10.26508/lsa.202000865 RRID:AB_1548761
B220 PE Validation: https://www.ncbi.nlm.nih.gov/pmc/articles/PMC6030061/ DOI: 10.1038/s41467-018-04999-8 RRID:AB_312993
F4/80 PE-Cy7 Validation: https://www.jimmunol.org/content/194/11/5529.long DOI: 10.4049/jimmunol.1403215
CD3e BV510 Validation: https://pubmed.ncbi.nlm.nih.gov/8986720/ DOI: 10.1016/s1074-7613(00)80275-7 RRID:AB_2737959
CD3 eFlour450 Validation: https://www.ncbi.nlm.nih.gov/pmc/articles/PMC7362728/ DOI: 10.3389/fimmu.2020.01397 RRID:AB_1272193
CD43 PE-Cy7 Validation: https://www.ncbi.nlm.nih.gov/pmc/articles/PMC6377614/ DOI: 10.1038/s41467-019-08637-9 RRID:AB_528813
CD27 BV510 Validation: https://www.ncbi.nlm.nih.gov/pmc/articles/PMC4258531/ DOI: 10.4049/jimmunol.1401702 RRID:AB_2565795
NK-1.1 PE/Dazzle™ 594 Validation: https://www.ncbi.nlm.nih.gov/pmc/articles/PMC6201245/ DOI: 10.1016/j.cell.2018.08.061 RRID:AB_2564219
NKp46 eFlour660 Validation: https://www.ncbi.nlm.nih.gov/pmc/articles/PMC5552247/ DOI: 10.1016/j.ebiom.2017.07.016 RRID:AB_10598664
CD11b Biotin Validation: https://pubmed.ncbi.nlm.nih.gov/7153706/ DOI: 10.1084/jem.156.4.1000 RRID:AB_394773
Ter119 Biotin Validation: https://pubmed.ncbi.nlm.nih.gov/1975515/ DOI: 10.1016/0092-8674(90)90262-d RRID:AB_394985
CD3e Biotin Validation: https://pubmed.ncbi.nlm.nih.gov/7528772/ DOI: 10.1084/jem.181.1.375 RRID:AB_394593
CD4 Biotin Validation: https://www.ncbi.nlm.nih.gov/pmc/articles/PMC2195704/ DOI: 10.1084/jem.190.10.1517 RRID:AB_394581
CD8 Biotin Validation: https://pubmed.ncbi.nlm.nih.gov/2140790/ DOI: doi: 10.1002/eji.1830200431 RRID:AB_394567
B220 Biotin Validation: https://pubmed.ncbi.nlm.nih.gov/11747358/ DOI: 10.1006/cimm.2001.1865 RRID:AB_394615
Streptavidin Pacific Orange Validation: https://pubmed.ncbi.nlm.nih.gov/19049809/ DOI: 10.1016/j.jim.2008.11.002
Sca1 Pacific Blue Validation: https://pubmed.ncbi.nlm.nih.gov/29438697/ DOI: 10.1016/j.ccell.2018.01.006 RRID:AB_2143237
CD48 AF700 Validation: https://pubmed.ncbi.nlm.nih.gov/32589864/ DOI: 10.1016/j.stem.2020.06.003 RRID:AB_10612755
CD150 PE-Cy7 Validation: https://pubmed.ncbi.nlm.nih.gov/23716557/ DOI: 10.3324/haematol.2012.080424 RRID:AB_439797
c-Kit APC Validation: https://www.ncbi.nlm.nih.gov/pmc/articles/PMC2213494/ DOI: 10.1084/jem.192.12.1707 RRID:AB_398536
c-Kit PE-CF594 Validation: https://pubmed.ncbi.nlm.nih.gov/7552996/ DOI: 10.1016/1074-7613(95)90116-7 RRID:AB_11154233
CD16/32 APC-Cy7 Validation: https://pubmed.ncbi.nlm.nih.gov/8406898/ DOI: 10.1128/iai.61.11.4925-4928.1993 RRID:AB_1645229
CD34 eFluor450 Validation: https://www.ncbi.nlm.nih.gov/pmc/articles/PMC6987315/ DOI: 10.3389/fendo.2019.00944 RRID:AB_2043837
CD34 eFluor660 Validation: https://www.ncbi.nlm.nih.gov/pmc/articles/PMC3572158/ DOI: 10.1371/journal.pone.0055890 RRID:AB_10596826
CD45.1 APC-Cy7 Validation: https://pubmed.ncbi.nlm.nih.gov/31932810/ DOI: 10.1038/s41590-019-0568-x RRID:AB_313505
CD45.2 APC-Cy7 Validation: https://pubmed.ncbi.nlm.nih.gov/33440149/ DOI: 10.1016/j.celrep.2020.108609 RRID:AB_830789
Ter119 BV510 Validation: https://www.ncbi.nlm.nih.gov/pmc/articles/PMC2556797/ DOI: 10.1084/jem.20080132 RRID:AB_2561661
CD51 PE Validation: https://www.ncbi.nlm.nih.gov/pmc/articles/PMC5754357/ DOI: 10.1038/s41467-017-02427-x RRID:AB_2129493
CD31 PE-CF594 Validation: https://pubmed.ncbi.nlm.nih.gov/9045924/ DOI: 10.1002/eji.1830270223 RRID:AB_2738320
CD140α APC Validation: https://www.ncbi.nlm.nih.gov/pmc/articles/PMC7835386/ DOI: 10.1038/s41419-021-03408-1 RRID:AB_529482
CD45.1 PE Validation: https://www.ncbi.nlm.nih.gov/pmc/articles/PMC5093323/ DOI: 10.1038/ncomms13180 RRID:AB_313497
CD45.2 APC Validation: https://pubmed.ncbi.nlm.nih.gov/19447103/ DOI: 10.1016/j.yexcr.2009.05.007 RRID:AB_389211
CD45.2 APC-Cy7 Validation: https://pubmed.ncbi.nlm.nih.gov/33440149/ DOI: 10.1016/j.celrep.2020.108609 RRID:AB_830789
CD45.1 Biotin Validation: https://pubmed.ncbi.nlm.nih.gov/31801071/ DOI: 10.1016/j.celrep.2019.10.111 RRID:AB_313493
CD45.2 Biotin Validation: https://pubmed.ncbi.nlm.nih.gov/32877667/ DOI: 10.1016/j.celrep.2020.108085 RRID:AB_313441
NK-1.1 Biotin Validation: https://www.ncbi.nlm.nih.gov/pmc/articles/PMC8257047/ DOI: 10.1016/j.celrep.2021.109264 RRID:AB_313391
CD19 Biotin Validation: https://www.ncbi.nlm.nih.gov/pmc/articles/PMC5707402/ DOI: 10.1038/s41598-017-16743-1 RRID:AB_313638

CD11b BB700 (PerCP Cy5.5) Validation: https://pubmed.ncbi.nlm.nih.gov/23610144/ DOI: 10.4049/jimmunol.1202270 RRID:AB_893232
CD193 PE Validation: https://pubmed.ncbi.nlm.nih.gov/33440150/ DOI: 10.1016/j.celrep.2020.108621 RRID:AB_2561533
CD170 (Anti-Siglec-F) BV421 Validation: https://www.ncbi.nlm.nih.gov/pmc/articles/PMC5045273/ DOI: 10.1016/j.bbrc.2016.08.055 RRID:AB_2810421
CD49b PE Validation: https://pubmed.ncbi.nlm.nih.gov/26511661/ DOI: 10.1038/ncomms9487 RRID:AB_313029
CD200R3 PE-Cy7 Validation: https://pubmed.ncbi.nlm.nih.gov/33450207/ DOI: 10.1016/j.cell.2020.12.033 RRID:AB_2814045
Anti-FcεRIα APC Validation: https://www.ncbi.nlm.nih.gov/pmc/articles/PMC6246036/ DOI: 10.1016/j.ccell.2018.09.003 RRID:AB_10640726
7-AAD BB700 (PerCP Cy5.5) Validation: https://pubmed.ncbi.nlm.nih.gov/22289926/ DOI: 10.1038/leu.2011.372
Zombie NIR™ Fixable Viability Kit APC-Cy7 Validation: https://pubmed.ncbi.nlm.nih.gov/25671696/ DOI: 10.1371/journal.pone.0115725
LIVE/DEAD™ Fixable Aqua Dead Cell Stain Kit Validation: https://pubmed.ncbi.nlm.nih.gov/22960222/ DOI: 10.1016/j.immuni.2012.06.011

Animals and other organisms

Policy information about studies involving animals; ARRIVE guidelines recommended for reporting animal research

Laboratory animals	Recipients for transplantations were purchased from Charles River (Ly5.1, B6.SJL PtprcaPepcb/BoyCrl). All recipient mice were male or female and 8-12 weeks old at the time of transplantation.<br><br>Cebpαcre/+ R26EYFP/+ SbdsF/+ mice were generated in house and were of C57/BL6 background<br><br>NK depletion experiments were conducted on six month old C57BL/6J male mice, also purchased from Charles River.<br><br>Animals were maintained in specific pathogen-free conditions in the Erasmus Animal Facility (EDC). Mice were housed in groups of maximum five animals (100cm2 per animal) under a standard 12 h light/dark cycle, with access to food and water ad libitum and an ambient temperature of 21-23 degrees Celsius with a humidity maintained between 40-70%. All mice were euthanized by cervical dislocation.
Wild animals	No wild animals were used in this study.
Field-collected samples	No field collected samples were used in this study.
Ethics oversight	Erasmus Animal Center (EDC)

Note that full information on the approval of the study protocol must also be provided in the manuscript.

Flow Cytometry

Plots

Confirm that:

- [x] The axis labels state the marker and fluorochrome used (e.g. CD4-FITC).
- [x] The axis scales are clearly visible. Include numbers along axes only for bottom left plot of group (a 'group' is an analysis of identical markers).
- [x] All plots are contour plots with outliers or pseudocolor plots.
- [x] A numerical value for number of cells or percentage (with statistics) is provided.

Methodology

Sample preparation	Bone marrow: Bones were crushed in 3x2x1mL of FACS buffer (PBS+0.5%FCS) with sterilized mortar and pestle. BM cells were pelleted by centrifuging, and BM supernatant was transferred to a clean Eppendorf tube and snap frozen in liquid nitrogen. BM cell suspension was passed through a 40µM filter. RBCs in BM were lysed in 2mL ACK lysing buffer (Lonza) for 4 min on ice, and washed in 20mL FACS buffer<br><br>Peripheral blood:PB was collected by cheek puncture monthly and at the end of follow-up periods. Complete blood count was measured on Scil Vet ABC Plus hematology analyzer (Scil Animal Care). Samples were stained with stated antibodies and lysed with IO test buffer conform manufacturer's protocol.<br><br>Bone Fraction: prepared by incubating bone fragments after crushing in 2mL 0.25% Collagenase Type I (07902, STEMCELL Technologies) for 45 min in 37°C water bath, with vortexing every 15 min, washed in 20mL FACS buffer and passed through a 40µM filter again
Instrument	All FACS analysis were performed on BD LSR II flow cytometer (BD Biosciences) and all cell sorting experiments were performed on BD FACSAria III flow cytometer (BD Biosciences).

Software	Data were processed with FlowJo software (Version 10.6.2)
Cell population abundance	Reanalyzing the sorted population. 100% purity.
Gating strategy	All gating strategy for each experiment were supplied in (supplementary) figures. Gating always started with mononuclear cell gating (based on FSC/SSC) to exclude debris. Subsequently doublet exclusion was done by SSC-W and SSC-A. Thereafter dead cells were excluded by protein or DNA binding markers followed by (a combination of) single color epitope-markers. All gating was controlled with single staining controls.

☒ Tick this box to confirm that a figure exemplifying the gating strategy is provided in the Supplementary Information.
