# Supplementary material for: Myeloid cells promote interferon signaling-associated deterioration of the hematopoietic system
Source: Nat Commun. 2022 Dec 10;13:7657. doi: 10.1038/s41467-022-35318-x (PMC9741615; doi:10.1038/s41467-022-35318-x)
Supplement: Supplementary file 3 — Description of Additional Supplementary Files [file 41467_2022_35318_MOESM3_ESM.pdf]

Description of Additional Supplementary Files

File name: Supplementary Data 1
Description: GSEA signatures (C2) found significantly enriched and depleted in neutropenic mice. NES, size, and FDR value of each gene set are as listed. GSEA: gene sets enrichment analysis. NES: normalized enrichment score. FDR: false discovery rate.
